# HLA‐dependent variation in SARS‐CoV‐2 CD8 ^+^ T cell cross‐reactivity with human coronaviruses

**DOI:** 10.1111/imm.13451

**Published:** 2022-03-07

**Authors:** Paul R. Buckley, Chloe H. Lee, Mariana Pereira Pinho, Rosana Ottakandathil Babu, Jeongmin Woo, Agne Antanaviciute, Alison Simmons, Graham Ogg, Hashem Koohy

**Affiliations:** ^1^ MRC Human Immunology Unit, Medical Research Council (MRC) Human Immunology Unit MRC Weatherall Institute of Molecular Medicine (WIMM) John Radcliffe Hospital University of Oxford Oxford UK; ^2^ MRC WIMM Centre for Computational Biology Medical Research Council (MRC) Weatherall Institute of Molecular Medicine John Radcliffe Hospital University of Oxford Oxford UK

**Keywords:** CD8 T cells, coronavirus, immunogenicity, peptide presentation, pre‐existing T cell immunity, SARS‐CoV‐2, T cell cross‐reactivity, T cell response

## Abstract

The conditions and extent of cross‐protective immunity between severe acute respiratory syndrome coronavirus 2 (SARS‐CoV‐2) and common‐cold human coronaviruses (HCoVs) remain open despite several reports of pre‐existing T cell immunity to SARS‐CoV‐2 in individuals without prior exposure. Using a pool of functionally evaluated SARS‐CoV‐2 peptides, we report a map of 126 immunogenic peptides with high similarity to 285 MHC‐presented peptides from at least one HCoV. Employing this map of SARS‐CoV‐2‐non‐homologous and homologous immunogenic peptides, we observe several immunogenic peptides with high similarity to human proteins, some of which have been reported to have elevated expression in severe COVID‐19 patients. After combining our map with SARS‐CoV‐2‐specific TCR repertoire data from COVID‐19 patients and healthy controls, we show that public repertoires for the majority of convalescent patients are dominated by TCRs cognate to non‐homologous SARS‐CoV‐2 peptides. We find that for a subset of patients, >50% of their public SARS‐CoV‐2‐specific repertoires consist of TCRs cognate to homologous SARS‐CoV‐2‐HCoV peptides. Further analysis suggests that this skewed distribution of TCRs cognate to homologous or non‐homologous peptides in COVID‐19 patients is likely to be HLA‐dependent. Finally, we provide 10 SARS‐CoV‐2 peptides with known cognate TCRs that are conserved across multiple coronaviruses and are predicted to be recognized by a high proportion of the global population. These findings may have important implications for COVID‐19 heterogeneity, vaccine‐induced immune responses, and robustness of immunity to SARS‐CoV‐2 and its variants.

## Graphical abstract

We identified 126 SARS‐CoV‐2 T cell targets that exhibit cross‐reactive potential with HCoV predicted peptide‐MHC. We found a subset of COVID‐19 patients whose SARS‐CoV‐2‐specific public TCRs are primarily directed toward these targets with cross‐reactive potential. For these patients, we found distinct HLA profiles. It is plausible that such patients may exhibit more robust protection against SARS‐CoV‐2 and its variants. Ten of the identified SARS‐CoV‐2‐HCoV peptides are highly conserved across multiple coronaviruses and are predicted to invoke T cell responses in high proportions of the global population, which is an encouraging insight in the search for pan‐coronavirus T cell targets.

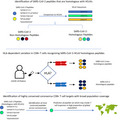

## INTRODUCTION

After almost 2 years, the severe acute respiratory syndrome coronavirus 2 (SARS‐CoV‐2) pandemic remains a global health challenge and causes a huge economic burden. SARS‐CoV‐2 virus gives rise to COVID‐19 disease, which is characterized by a heterogeneous clinical outcome ranging from asymptomatic infection to severe acute respiratory distress and death. The virus has proven to be dynamic, and the emergence of ‘variants of concern’ (e.g. the delta variant) challenges the existing mitigation strategies including vaccine rollouts [1].

Although disease morbidity is associated with several factors including age, sex and aberrant immune response, the mechanisms and factors underpinning the heterogeneity of disease are incompletely understood [2]. Furthermore, reports of differential immune responses following vaccination have started to emerge, demonstrating prior SARS‐CoV‐2 infection can enhance COVID‐19 vaccine response compared with naïve individuals [3, 4]. Despite the great recent efforts, many questions regarding the magnitude and robustness of immune response in disease, variants of concern and/or COVID‐19 vaccination in different individuals, remain open.

In particular, the extent of T cell cross‐reactivity between SARS‐CoV‐2 and other viruses and its impact on COVID‐19 disease is incompletely understood. Since Don Mason's seminal work on the existence of T cell cross‐reactivity [5]; its extent [6] and importance in T cell recognition have been extensively studied, and thus recognized as an essential feature of T cell responses [6, 7, 8, 9]. As such, the role and involvement of T cell cross‐reactivity have been widely investigated in multiple human diseases, including cancer [10, 11, 12], auto‐immune disease [13, 14, 15], infections [16, 17, 18, 19] such as dengue [20, 21] and zika [22], and unsurprisingly for SARS‐CoV‐2 [23, 24, 25, 26].

Several studies [23, 27] have illustrated that the correlates of immunity to SARS‐CoV‐2 are implicated by the presence of pre‐existing immunological memory conferred from cross‐reactivity to other viruses. On the contrary, such cross‐reactivity could modulate disease severity, vaccine response and/or protection against SARS‐CoV‐2 and its variants via the presence of antigen‐specific memory T cells [28]. Conversely, cross‐reactivity may provoke immunopathology through mechanisms such as antibody‐dependent enhancement of infection, with the potential for virus‐induced autoimmune disease in years to come [16, 29, 30].

Coronavirus strains that infect humans belong to either alpha or beta genera. The alphacoronaviruses contain HCoV‐229E and ‐NL63 while the four lineages of betacoronaviruses include HCoV ‐OC43 and ‐HKU1, SARS‐CoV and ‐CoV‐2, MERS‐CoV and other viruses only identified in bats. HCoV‐OC43, ‐HKU1, ‐NL63 and ‐229E strains are known to cause mild to moderate ‘common cold’ symptoms whereas MERS‐, SARS‐CoV‐1 and ‐2 can cause severe respiratory tract disease and death. Previous natural and experimental infection studies in humans suggest antibody cross‐reactivity within—but minimal reactivity between—endemic human alpha and beta coronaviruses. Unlike antibodies, T cell cross‐reactivity to SARS‐CoV‐2 appears to be more prevalent. Several recent studies have reported the existence of SARS‐CoV‐2‐specific T cells in unexposed individuals [24, 31, 32, 33, 34, 35, 36], although it appears that T cell cross‐reactivity is more pronounced in CD4 ^+^ than CD8 ^+^ T cells in these subjects.

Recent studies have provided varying insights regarding the presence of pre‐existing CD8 ^+^ T cell immunity to SARS‐CoV‐2 conferred by HCoV. In an investigation into the immunodominant SARS‐CoV‐2 SPR* epitope—associated with HLA‐B*07:02—Nguyen et al [37], found little evidence of cross‐reactive exposure in pre‐pandemic Australian samples. On the contrary, Francis et al [36]. found evidence of pre‐existing memory CD8 ^+^ T cells in naïve samples and have shown that HLA genotype conditions pre‐existing CD8 ^+^ T cell memory to SARS‐CoV‐2, and they suggest that unexposed individuals with specific HLA alleles (such as HLA‐B*07:02), may be more likely to possess cross‐reactive memory T cells specific for the SPR* SARS‐CoV‐2 epitope. These disparate results may stem from differences in regional HLA allele frequencies and/or experimental methodology. Nevertheless, the extent to which patients’ haplotypes and SARS‐CoV‐2‐HCoV cross‐reactivity—amongst other factors—are linked to heterogeneous COVID‐19 disease, the robustness of immunity against SARS‐CoV‐2 and its variants, and/or protection after vaccine‐induced immune response, remains to be elucidated.

In this study, we examined the evidence for SARS‐CoV‐2‐specific T cell cross‐reactivity with common‐cold HCoVs and identified 126 immunogenic SARS‐CoV‐2 peptides that are highly similar to 285 predicted HCoV pMHC. We additionally identified a set of SARS‐CoV‐2 peptides with high similarity to several human proteins. We found that public TCR repertoires reactive to SARS‐CoV‐2 in COVID‐19 patients who carry specific HLA alleles primarily recognize SARS‐CoV‐2 peptides with high similarity to HCoVs, suggesting that common‐cold HCoV cross‐reactivity is variable and likely to be conditioned by HLA. It is plausible that patients carrying these HLAs may exhibit more robust protection against SARS‐CoV‐2 and its variants. We lastly identified a set of 10 peptides that are highly conserved across multiple coronavirus strains, to serve not only as potential pan‐coronavirus T cell targets, but we propose are leading candidates as cross‐reactive CD8 ^+^ T cell epitopes.

## RESULTS

### Curation of functionally evaluated SARS‐CoV‐2 peptides

**FIGURE 1:**
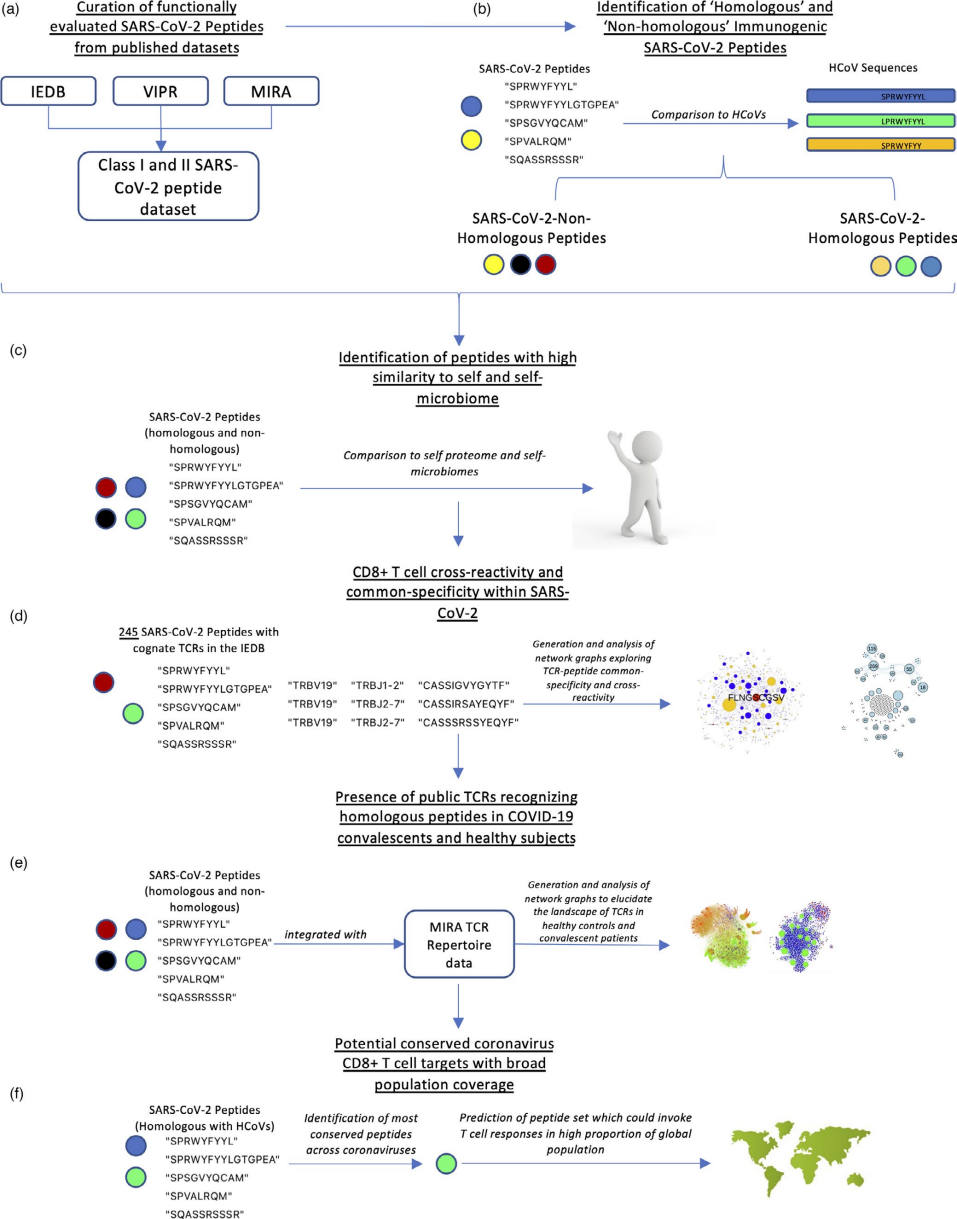
Overview of the study. (a) An illustration describing the curation of a SARS‐CoV‐2 peptide immunogenicity dataset from published data. (b) A map of potential cross‐reactive SARS‐CoV‐2 peptides and their target counterparts from HCoVs is presented. (c) A set of immunogenic SARS‐CoV‐2 peptides with highsimilarity to the human proteome is reported. (d) The extent of cross‐reactivity and common‐specificity within SARS‐CoV‐2 is examined. (e) The landscape of potential SARS‐CoV‐2‐specific cross‐reactive public TCRs in health and/or COVID‐19 disease is detailed. (f) A set of 10 peptides which are highly conserved across HCoVs, SARS, SARS‐CoV‐2 and MERS are predicted to exhibit high global and regional ‘population coverage’ is identified

**FIGURE 2:**
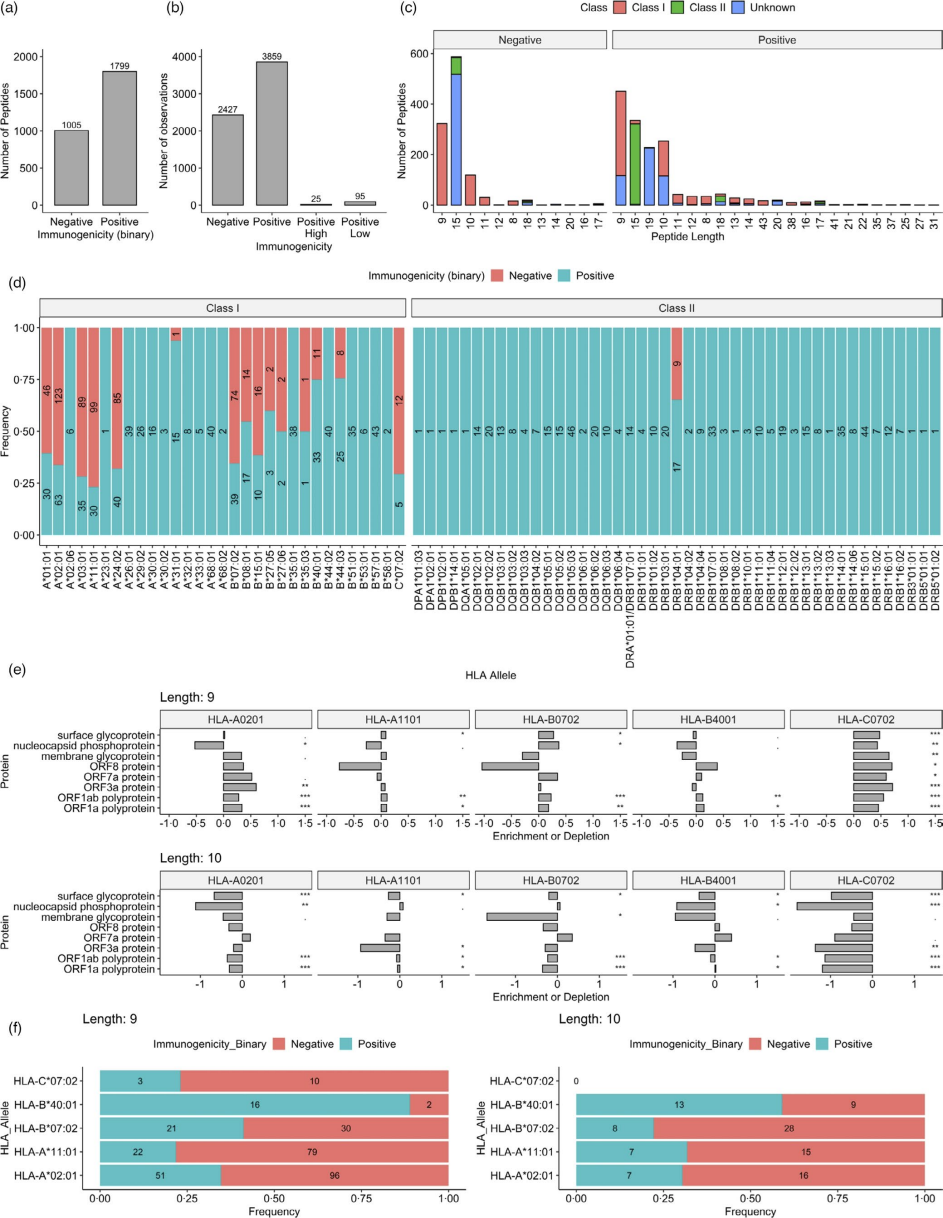
A comprehensive pool of functionally validated SARS‐CoV‐2 peptides. Barplots showing (a) The number of SARS‐CoV‐2 peptides deemed’positive’ or ‘negative’. ‘Negative’ label reflects only negative qualitative observations and ‘positive’ reflects at least one immunogenic observation. (b) The number of total observations, including all assay and HLA combinations for each peptide. (c) The distribution of lengths of unique observations (peptide‐immunogenicity) in our SARS‐CoV‐2 dataset. Left panel shows nonimmunogenic ‘Negative’ peptides. Right panel shows immunogenic or ‘Positive’ peptides. MHC class of the unique observation is colour coded. (d) The frequencies of total immunogenic or non‐immunogenic observations (peptide‐MHC‐immunogenicity), where a specific HLA allele is available for a peptide. Numeric labels show the number of peptides in each group. (e) The logs odd ratio of observed and expected number of presented peptides of lengths 9 and 10 by common HLA alleles for SARS‐CoV‐2 proteins >100 amino acids in length. Significance calculated using binomial distribution. (f) The frequency of immunogenic and nonimmunogenic peptides as presented by HLA alleles arising from SARS‐CoV‐2. Numeric labels show the number of observations per immunogenicity status

To investigate the potential for T cell cross‐reactivity against SARS‐CoV‐2 conferred by common‐cold HCoVs, we curated a comprehensive pool of SARS‐CoV‐2 class I and II peptides from three previously published datasets (see Methods), which have been functionally evaluated for CD4 ^+^ and CD8 ^+^ T cell responses (Figure 1: study overview). The data comprise 1799 and 1005 immunogenic and non‐immunogenic SARS‐CoV‐2 *peptides*, respectively (Figure 2a). Many of these peptides were tested for T cell reactivity in the context of multiple HLA alleles and/or by multiple assays (IFNγ, IL‐5 production, etc.). Furthermore, some peptides are described by qualitative labels corresponding to varying response magnitude (Positive‐high and Positive‐low, etc.). Taking various combinations of peptides and MHC molecules into account, we found 3979 and 2427 immunogenic and non‐immunogenic observations (Figure 2b). For unique immunogenic observations, the most common lengths are 9 mers, followed by 15 and 10 mers (Figure 2c), and of the total immunogenic observations 36·0% are presented by class I MHC, 32·9% by class II (Figure 2d) and for 31% MHC type is unknown (Figure S1a). For non‐immunogenic observations, 36·1% are presented by class I, 26·4% by class II and for 37·51% the MHC is unknown. At the gene level, HLA‐allele specific information was available for 934 (56·5%) and 607 (42·2%) of immunogenic class I and II observations, respectively (Figure S1a).

Given the high proportion of missing MHC information, we employed netMHCpan 4.1 and netMHCIIpan to predict presenting class I and class II alleles respectively for immunogenic peptides (see Methods). Here, we were able to identify 98% of known MHC molecules, providing confidence in predictions for unknown alleles (Figure S1b).

We next sought to examine whether HLAs exhibit preferences towards presenting peptides from certain SARS‐CoV‐2 proteins. By employing a similar methodology to Karnaukhov et al [38], we gauged the enrichment and depletion of HLA ligands arising from these proteins (see Methods). Indeed, we observed differential antigen presentation by HLAs e.g. HLA‐C*07:02 appears to be the most consistently enriched in presenting 9mers from the examined proteins (Figure 2e), while HLA‐A*02:01 is enriched in presenting 9mers from ORFs but depleted for 10mers across most assessed proteins. This disparity may be due to a known preference of 9 mers for HLA‐A*02:01 [39]. Furthermore, despite the prevalence of HLA A*02:01 in the global population and in the MHC presentation experiments, this allele appears to be depleted for presenting ligands from SARS‐CoV‐2 proteins that have been the focus of intense experimental work, e.g. spike and nucleocapsid phosphoprotein.

These patterns of HLA preferences in presenting SARS‐CoV‐2 peptides appear to differ for 9 and 10 mers. For example, whereas HLA‐C*07:02 is enriched for presenting 9 mers, this allele appears to be a poor presenter of 10mers from each examined protein. It is unclear why substantially fewer 10 mer HLA‐C*07:02 ligands are predicted than 9mers, however, it is plausible that this allele may prefer 9mers, as appears to be the case with HLA‐A*02:01, ‐A*11:01 and ‐B*40:01 [39], or that this may be a SARS‐CoV‐2 specific effect.

Although it is of great interest to reveal the rate at which SARS‐CoV‐2 MHC‐bound peptides are immunogenic in humans [40], it cannot be examined directly with existing data because not all MHC‐bound SARS‐CoV‐2 peptides have been evaluated for immunogenicity. Nevertheless, we explored the pool of MHC‐bound peptides in our dataset that have been examined for a T cell response, to gauge the proportion that SARS‐CoV‐2 pMHC are immunogenic. Overall, we observed low rates of immunogenic pMHC (Figure 2f), although ligands of HLA‐B*40:01 appear to be commonly immunogenic. Interestingly, we observed that HLA‐C*07:02 does not present any 10 mers in our dataset. This apparent preference for 9 mers is consistent with the availability of HLA‐C*07:02 ligands tested for T cell response in humans from the IEDB, where there exist only 121 unique peptides, of which 73% are 9 mers and only 12% are 10 mers. In summary, these data suggest length and source protein preferences for HLA alleles presenting SARS‐CoV‐2 peptides and that HLA‐B*40:01 SARS‐CoV‐2 ligands are commonly immunogenic.

### Identification of Homologous and Non‐homologous Immunogenic SARS‐CoV‐2 peptides

To discriminate SARS‐CoV‐2‐HCoV homologous (hereby referred to as ‘SARS‐CoV‐2‐HCoV’) peptides, we compared immunogenic SARS‐CoV‐2 peptides to HCoV protein sequences. For this, we defined a metric that considers (1) sequence homology, (2) physicochemical similarities (MatchScore [41]) and (3) presentation status for which the source peptide from SARS‐CoV‐2 and the target peptide from one of the HCoVs are required to be presented by the same HLA. A source peptide is defined as ‘homologous’ if it fulfils all these three conditions, otherwise, it is considered a ‘non‐homologous’ peptide (see Methods).

Using our metric, we identified 126 unique SARS‐CoV‐2 (immunogenic) peptides pointing to 285 highly similar peptides in HCoVs (Data File S1). Hence, we provide a comprehensive map of non‐homologous and homologous SARS‐CoV‐2 functionally evaluated immunogenic peptides, and for SARS‐CoV‐2‐HCoV peptides, their matches from each HCoV.

**FIGURE 3:**
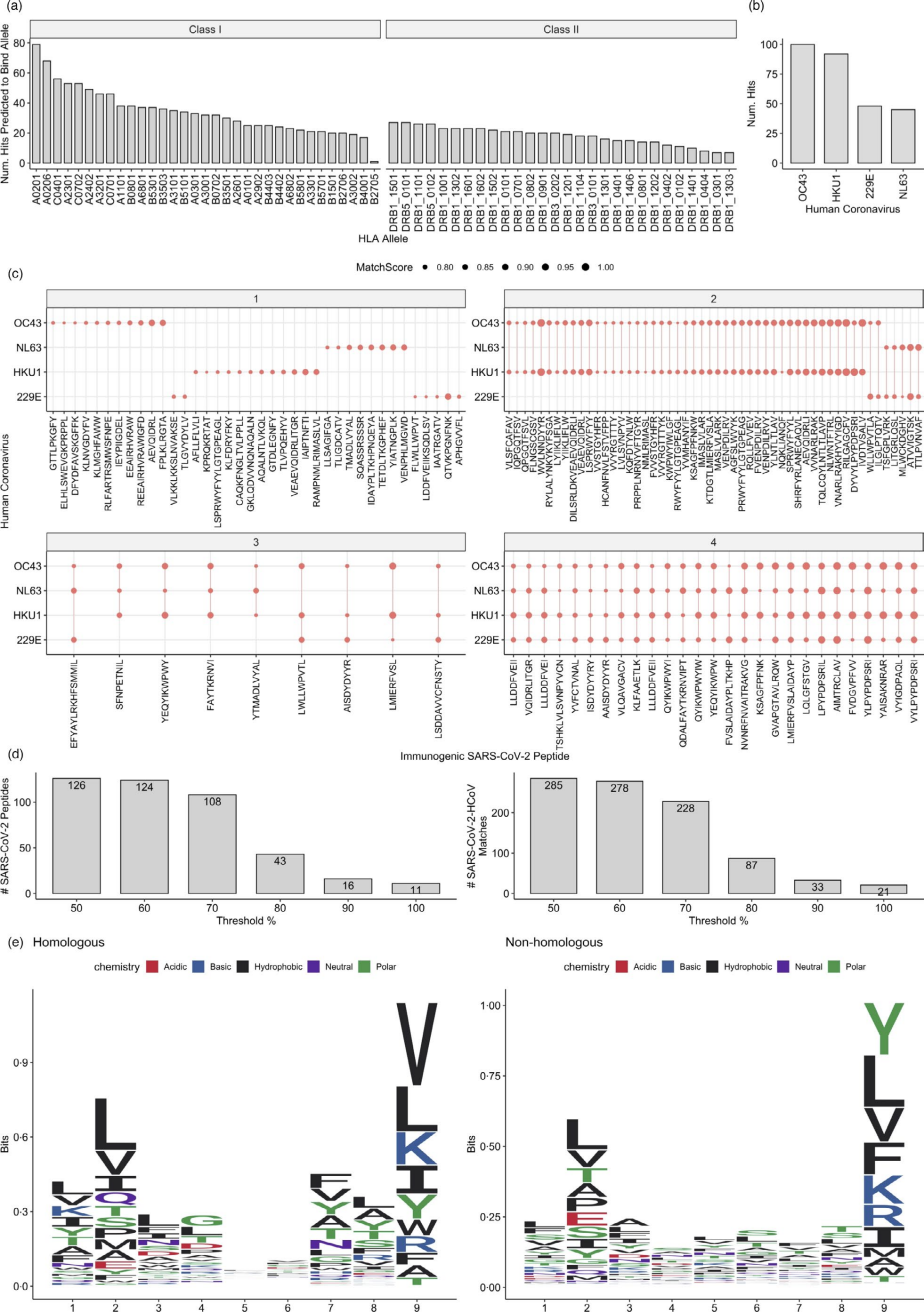
A set of peptides from human coronavirus strains with high similarity to immunogenic SARS‐CoV‐2 peptides. (a) A barplot showing the number of high similarity matches predicted to bind a set of common HLA class I and class II alleles. (b) A barplot showing the number of unique high similarity matches derived from each human common‐cold‐causing coronavirus. Each hit is defined as a unique observation—that fulfills all three criteria defined in our similarity metric—between an immunogenic SARS‐CoV‐2 peptide with length *l*, and a stretch of length*l*from one viral protein. (c) A dot and line plot showing each SARS‐CoV‐2 peptide and to which common‐cold‐causing coronavirus it exhibits a high similarity match. The size of each point represents the MatchScore, which reflects the physicochemical similarity with a counterpart HCoV match. Peptides are grouped by the number of high similarity matches one exhibits to human coronavirus strains (either 1, 2, 3 or 4). (d) Barplots showing the number of unique SARS‐CoV‐2 peptides (left) and SARS‐CoV‐2‐HCoV matches (right), at different thresholds of the sequence homology metric, i.e the % of the amino acids that must be conserved between the SARS‐CoV‐2 peptide and its HCoV match. (e) Sequence logo plots comparing amino acid usage of SARS‐CoV‐2‐homologous and non‐homologous 9‐mer peptide sequences

Out of the HLAs tested (see Methods) 33 and 28 class I and II HLAs, respectively, were predicted to present the target HCoV pMHCs (Figure 3a). HLA‐A*02:01 and HLA‐B*27:05 were the most and least common class I presenters, respectively. For class II, DRB1‐1501 and DRB5‐0101 were the most common presenters, while DRB1‐0301 and DRB1‐1303 were the least. Most homologous class I and II peptides were predicted to bind multiple HLA allelic variants (Figure S2a). Compared with non‐homologous peptides it appears that SARS‐CoV‐2‐HCoV peptides are presented by less HLAs, although this was not significant (Figure S2c). Nevertheless, the range of predicted alleles for these peptides suggests recognition in broad geographical and ethnic settings [42].

For the 126 SARS‐CoV‐2 peptides with high similarity to HCoV, we also observed binding to multiple HLAs (Figure S2b). In addition, we found that 9 mers comprise 54% of the 126 SARS‐CoV‐2 peptides with high‐similarity matches to HCoV, followed by 15 mers (19%) and 10 mers (17·5%) (Figure S2c). Consistent with previous reports [43], the betacoronaviruses HKU1 and OC43 were most enriched in target matches (Figure 3b), perhaps due to higher total sequence homology among betacoronavirus strains [25]. We next examined the extent to which immunogenic SARS‐CoV‐2 peptides exhibit homology to *multiple* HCoV strains. Surprisingly, we found that 36 SARS‐CoV‐2 immunogenic peptides were homologous to at least three strains (Figure 3c). However, we observed small clusters of peptides that only possess homology with one strain, e.g. OC43 or HKU1. ORF1ab protein and surface glycoprotein (spike) produced the highest quantity of homologous SARS‐CoV‐2‐HCoV peptides in both strains, and the protein regions from which these peptides were found are similar in both HKU1 and OC43 (Figure S2e–h).

Of particular note is that this map of homologous and non‐homologous peptides is subject to thresholds that we used in our similarity metric. The *sequence homology* threshold that was employed here is 50%, although most SARS‐CoV‐2‐HCoV peptides had greater than or equal to 70% sequence homology with their counterpart matches from HCoVs (Figure S2e). While a more stringent sequence homology parameter will produce a map containing fewer homologous peptides (Figure 3d), our main conclusions in this manuscript remain the same even with a sequence homology threshold of 70% (data not shown).

Lastly, we compared the amino acid distribution between homologous and non‐homologous SARS‐CoV‐2 peptides for 9 mers, which is the most common peptide length in our dataset (Figure 3e). We observed some moderate differences, e.g. increased prominence of Valine at position 9 within homologous peptides.

We have therefore identified a pool of 126 SARS‐CoV‐2 immunogenic peptides—that exhibit high similarity to 285 peptides in HCoV strains—which are likely to be presented by an array of class I and II HLA molecules. This array of presenting alleles suggests the potential for broad global population coverage, which is explored later. We propose that this pool of experimentally confirmed immunogenic SARS‐CoV‐2 peptides and their counterpart high similarity matches be considered as potential targets for T cell cross‐reactivity, therefore warranting investigation into pre‐existing immune memory from HCoV or a role in protection from SARS‐CoV‐2 variants.

### Identification of peptides with high similarity to self and self‐microbiomes

To prevent aberrant T cell‐mediated inflammation and tissue damage, the immune system has evolved several checkpoint mechanisms. These include thymus negative selection and peripheral tolerance. Indeed, dissimilarity to self is increasingly recognized as a component of peptide immunogenicity [44], which may assist in calibrating a balance between immunogenicity and inflammatory pathogenesis.

**FIGURE 4:**
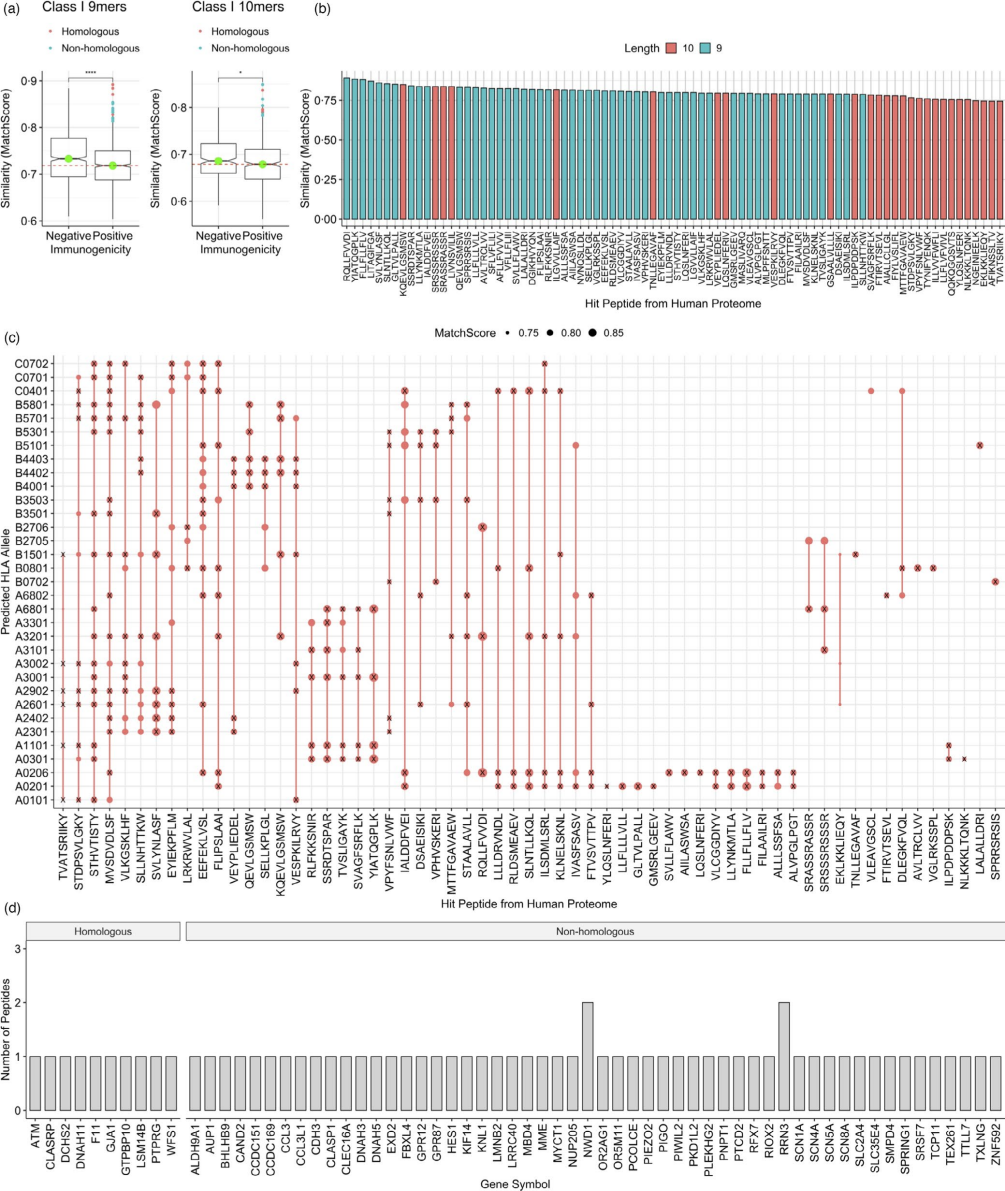
A pool of immunogenic SARS‐CoV‐2 peptides with high similarity to human genes. (a) Jittered boxplots showing the similarity—evaluated by the ‘MatchScore’—of nonimmunogenic and immunogenic SARS‐CoV‐2 peptides with sequences derived from the human proteome of lengths 9 (immunogenic *n* = 906, nonimmunogenic *n* = 734 peptides) and 10 (immunogenic *n* = 394 and nonimmunogenic *n* = 394 peptides). The green dot indicates the median of each group, red line shows the median of the immunogenic group. (b) A barplot showing the physicochemical similarity scores (MatchScores) of peptides from the human proteome which were found to have high similarity with immunogenic SARS‐CoV‐2 peptides, labelled by protein length. (c) A dotplot showing the predicted HLAs of peptides from the human proteome which exhibit ‘high similarity’ to SARS‐CoV‐2 proteome. The size of the point reflects the MatchScore. An ‘X’ shows where the SARS‐CoV‐2 derived peptide and the match are predicted to bind the same allele. (d) A bar chart showing the genes from which the high similarity match peptides arise from the human proteome, grouped by whether the SARS‐CoV‐2 peptide exhibits a high similarity match with HCoV (Homologous) or not (Nonhomologous)

To evaluate the extent to which dissimilarity to self and self‐microbiomes contribute to SARS‐CoV‐2 peptide immunogenicity, we took a similar approach and used our metric to compare SARS‐CoV‐2 peptides to human self‐proteome and microbiomes that include 457 gut and 50 airway microbiota (see Methods). Here, for SARS‐CoV‐2 HLA class I presented 9 and 10 mer peptides—for which we had the highest number of class I peptides—we observed that immunogenic SARS‐CoV‐2 peptides were significantly more dissimilar to the human proteome than their non‐immunogenic counterparts (Figure 4a and Figure S3a). Using this approach, we could not detect any significant difference between immunogenic and non‐immunogenic class II peptides in their dissimilarity to self‐proteome (Figure S3b).

Interestingly, however, for peptides of both lengths 9 and 10, we identified several immunogenic SARS‐CoV‐2 peptides with considerable sequence similarity to the human proteome (Figure 4a,b, Table S1). For the top 10% of these peptides with the highest similarity to self, the mean amino acid conservation (the proportion of the amino acid sequence which is exactly conserved) between these peptides and corresponding self‐peptides is 72·1% with an 8·33% standard deviation (see Data File S2 for the number of substitutions under column ‘Hamming’). In general, T cells specific for these peptides should be subject to negative selection otherwise it is plausible that aberrant immune responses may occur during the course of the disease in the form of immunopathology or the future in the form of autoimmunity [29, 45, 46].

To investigate the potential association of these peptides in immunopathology further, we predicted MHC presentation by a set of class I HLA alleles (see Methods) for the top 10% of peptides most similar to the human proteome for 9mers and 10mers. We observed that these peptides with high similarity to self are predicted to bind multiple HLAs (Figure 4c), and interestingly, we found that in most cases, the SARS‐CoV‐2 immunogenic peptide and the match from the human proteome are predicted to be presented by the same allele (Figure 4c).

Next, we examined the list of genes with high sequence similarities to these SARS‐CoV‐2 immunogenic peptides (Table S1 and Data File S2). Of particular interest, we found e.g. CCL3 and CCL3L1 which are linked to cytokine storms and the expression of which have been reported to be elevated in severe COVID‐19 patients [47, 48, 49, 50, 51, 52] (Figure 4d, Data File S2). We additionally observed CD163, similarly associated with severe COVID‐19, however, the predicted presentation score of HLA‐B*15:01 for peptides from CD163 with high similarity to SARS‐CoV‐2 was slightly beyond the generally accepted ‘binding’ cut‐off. Interestingly, the SARS‐CoV‐2 peptides which exhibited sequence similarity with CCL3 and CCL3L1 (and CD163) were not homologous with HCoVs (Figure 4d and Table 1)—which may increase the likelihood of being involved in immunopathology after infection. Additionally, we observed considerable amino acid conservation with matches from these genes, with 77·8% for 9 mers and 70% for 10 mers (Table 1).

**TABLE 1:** SARS‐CoV‐2 peptides with high similarity to the human proteome, from genes reported to be involved with severe COVID‐19. Note, peptides derived from CD163 were not predicted to bind HLA

Human match	MatchScore	SARS‐CoV−2 Peptide	HLA Allele	Homologous/Non‐homologous	Protein	Length	Proportion conserved
STAALAVLL	0·806	STAALGVLM	HLA‐A*26:01	Non‐homologous	>sp|P10147|CCL3_HUMAN C‐C motif chemokine 3 OS=Homo sapiens OX=9606 GN=CCL3 PE=1 SV=1	9	0·778
STAALAVLL	0·806	STAALGVLM	HLA‐A*26:01	Non‐homologous	>sp|P16619|CL3L1_HUMAN C‐C motif chemokine 3‐like 1 OS=Homo sapiens OX=9606 GN=CCL3L1 PE=1 SV=1	9	0·778
ILGVVLLAIF	0·818	IVGVALLAVF	HLA class I	Non‐homologous	>sp|Q86VB7|C163A_HUMAN Scavenger receptor cysteine‐rich type 1 protein M130 OS=Homo sapiens OX=9606 GN=CD163 PE=1 SV=2	10	0·7
ILGVVLLAIF	0·818	IVGVALLAVF	HLA class I	Non‐homologous	>tr|F5GZZ9|F5GZZ9_HUMAN Scavenger receptor cysteine‐rich type 1 protein M130 OS=Homo sapiens OX=9606 GN=CD163 PE=1 SV=1	10	0·7
ILGVVLLAIF	0·818	IVGVALLAVF	HLA class I	Non‐homologous	>tr|H0YFM0|H0YFM0_HUMAN Scavenger receptor cysteine‐rich type 1 protein M130 (Fragment) OS=Homo sapiens OX=9606 GN=CD163 PE=1 SV=1	10	0·7
ILGVVLLAIF	0·818	IVGVALLAVF	HLA class I	Non‐homologous	>tr|C9JHR8|C9JHR8_HUMAN Scavenger receptor cysteine‐rich type 1 protein M130 OS=Homo sapiens OX=9606 GN=CD163 PE=1 SV=1	10	0·7

CCL3 and CCL3L1 are both ligands for CCR1 and CCR5. Interestingly, CCR1 variants are linked to pulmonary macrophage infiltration in severe COVID‐19 [53] and inhibition of CCR5 in critical COVID‐19 patients has been associated with a decrease in plasma IL‐6 and SARS‐CoV‐2 RNA and an increase in CD8 ^+^ T cells [54]. Additionally, intermediate monocytes which constitutively express high levels of CCR5 have recently been suggested as playing a role in post‐acute sequelae of COVID‐19 [55] (often referred to as ‘long‐COVID’). Of further interest, we found SMPD4 and SLC1A4, which together with CCL3 and CCL3L1 are involved in the response to TNF, which is part of the cytokine storm following COVID‐19 disease.

By comparing SARS‐CoV‐2 peptides to human microbiomes, we observed subtle higher dissimilarity of SARS‐CoV‐2 immunogenic peptides to the gut (Figure S3c) and airways (Figure S3d) microbiomes, which may suggest a link between the diversity of both microbiota and heterogeneity of the disease in populations, although this warrants further investigation.

Given the magnitude of the global pandemic and the widespread vaccination required to combat it, future virus‐induced autoimmune disease and immunopathology is of concern. Overall, this analysis suggests dissimilarity of viral peptides to self‐proteins as a correlate of peptide immunogenicity. Furthermore, we present candidate genes and peptides with high similarity to SARS‐CoV‐2 T cell targets, which we suggest as prime targets for further investigations into their role in autoimmune disease and immunopathology following SARS‐CoV‐2 infection and/or vaccination.

### CD8 ^+^ T cell cross‐reactivity and common‐specificity within SARS‐CoV‐2

A valuable characteristic of our map of SARS‐CoV‐2‐HCoV homologous and non‐homologous peptides is that for 245 of these (out of 1279 class I immunogenic peptides), cognate TCRs at the beta chain resolution are available in the IEDB. We, therefore, set out to map the TCR landscape through a network approach to explore the potential for cross‐reactivity among SARS‐CoV‐2‐specific CD8 ^+^ T cells and their common specificity. Here, to avoid overestimating connectivity, any peptides of different lengths, which share starting positions in the SARS‐CoV‐2 proteome and are recognized by identical sets of TCRs, are considered as one peptide.

Through a two‐mode (bipartite) network‐graph illustrating the connectivity of SARS‐CoV‐2 immunogenic peptides with their cognate TCRs, amongst a highly connected topography we observed considerable connectivity for some SARS‐CoV‐2‐HCoV peptides e.g. ‘FLN’ (Figure S4a). Exploring this further, we projected the bipartite network graph into a one‐mode graph where nodes represent peptides and an edge between two nodes requires the existence of a TCR recognising both peptides (Figure S4b). The clustering around a small set of hubs suggests that many experimentally assessed TCRs target a small set of SARS‐CoV‐2 peptides. Indeed, we found that in this dataset, 80% of the TCRs are reported to recognize only 40 (16%) peptides, of which 4 are SARS‐CoV‐2‐HCoV peptides and 36 are non‐homologous (Figure S4c). This dominant set of peptides may be due to experimental biases e.g. research may be heavily biased toward several protein regions. However, this may also reflect a selection bias by SARS‐CoV‐2 specific TCRs. In this regard, amongst the TCRs recognising these dominant peptides, we observed high usage of V gene TRBV20‐1 [56] and J gene TRBJ2‐1 [57] (Figure S4d), which have been previously reported to have implications in COVID‐19 patients.

Similarly, we examined the extent of common specificity in SARS‐CoV‐2 specific T cells by a one‐mode graph in which nodes represent TCRs and an edge represents whether two nodes (TCRs) recognize the same peptide (Figure S4e). Interestingly, this graph reveals a set of highly connected hubs reflecting levels of common specificity, however, there are many TCRs that recognize only a single unique peptide. Comparing these two sets of TCRs, we did not observe considerable differences in their CDR3β sequences (Figure S4f–g), however, we observed differences in V and V‐J gene usage (Figure S4h–j).

In summary, we employed peptides with known cognate TCRs in the IEDB database—although limited in numbers—to explore SARS‐CoV‐2 CD8 ^+^ T cell cross‐reactivity. Our network approach demonstrates that SARS‐CoV‐2 CD8 ^+^ T cells can cross‐react and exhibit common specificities.

### Presence of public TCRs recognising SARS‐CoV‐2‐HCoV peptides in COVID‐19 convalescents and healthy subjects

We next integrated our map of SARS‐CoV‐2 homologous and non‐homologous peptides with a recently published dataset known as ‘MIRA’ [58] to track the patterns of public TCRs (defined as CDR3β+V + J gene(s) present in at least two subjects) recognizing SARS‐CoV‐2‐HCoV peptides in convalescents and/or healthy subjects. Here, Nolan et al., employed the multiplex identification of antigen‐specific T cell receptors (MIRA) assay to identify SARS‐CoV‐2 specific TCRs from PBMCs and naïve T cells. These data include more than 160k high confidence SARS‐CoV‐2‐specific TCRs mapped to target peptides from 39 healthy controls (HC) (defined as unexposed to SARS‐CoV‐2) and 90 COVID‐19 convalescent patients. These data consist of 792 unique SARS‐CoV‐2 peptides, 54 of which are SARS‐CoV‐2‐HCoV homologous peptides.

First, we set out to identify any shared biochemical features of these public TCRs which recognize only SARS‐CoV‐2‐HCoV peptides compared with those which recognize only non‐homologous SARS‐CoV‐2 peptides [56, 59, 60, 61, 62]. Between these two groups, we observed only minor differences in CDR3 motifs and lengths of these sequences, for which the effect of technical variation could not be ruled out (Figure S5a–d). We did not observe any evidence of J gene bias (Figure S5e), although we did observe some differences in V gene usage, in particular for TCRBV‐5–01 (Figure S5e), as well as a potential bias toward TCRBV05‐01‐TCRBJ‐02–01 usage (Figure S5g,h).

Next, we examined whether any of the public TCRs in the MIRA dataset that recognize SARS‐CoV‐2‐HCoV peptides were reported to react with epitopes from other viruses, which would suggest a level of cross‐reactivity of these TCRs. Interestingly, by comparing the CDR3βs in the MIRA dataset with those in VDJdb [63] recognising epitopes from other viruses, we observed some SARS‐CoV‐2‐specific TCRs which recognize peptides from CMV, Influenza A, EBV, HIV‐1 and Homo sapiens (Figure S6a) suggesting some elements of cross‐reactivity with other pathogens (Data File S3). We observed minor differences in the motifs of the SARS‐CoV‐2‐specific CDR3βs which cross‐react with CMV (Data File S6b), Influenza A (Data File S6c) and EBV (Data File S6d), although this may only reflect differences in the CDR3s which recognize these different viruses. Indeed, it is also important to note that the use of the MIRA dataset for this particular type of analysis has limitations, and more robust conclusions regarding cross‐reactivity to other pathogens would require more SARS‐CoV‐2‐specific T cells.

**FIGURE 5:**
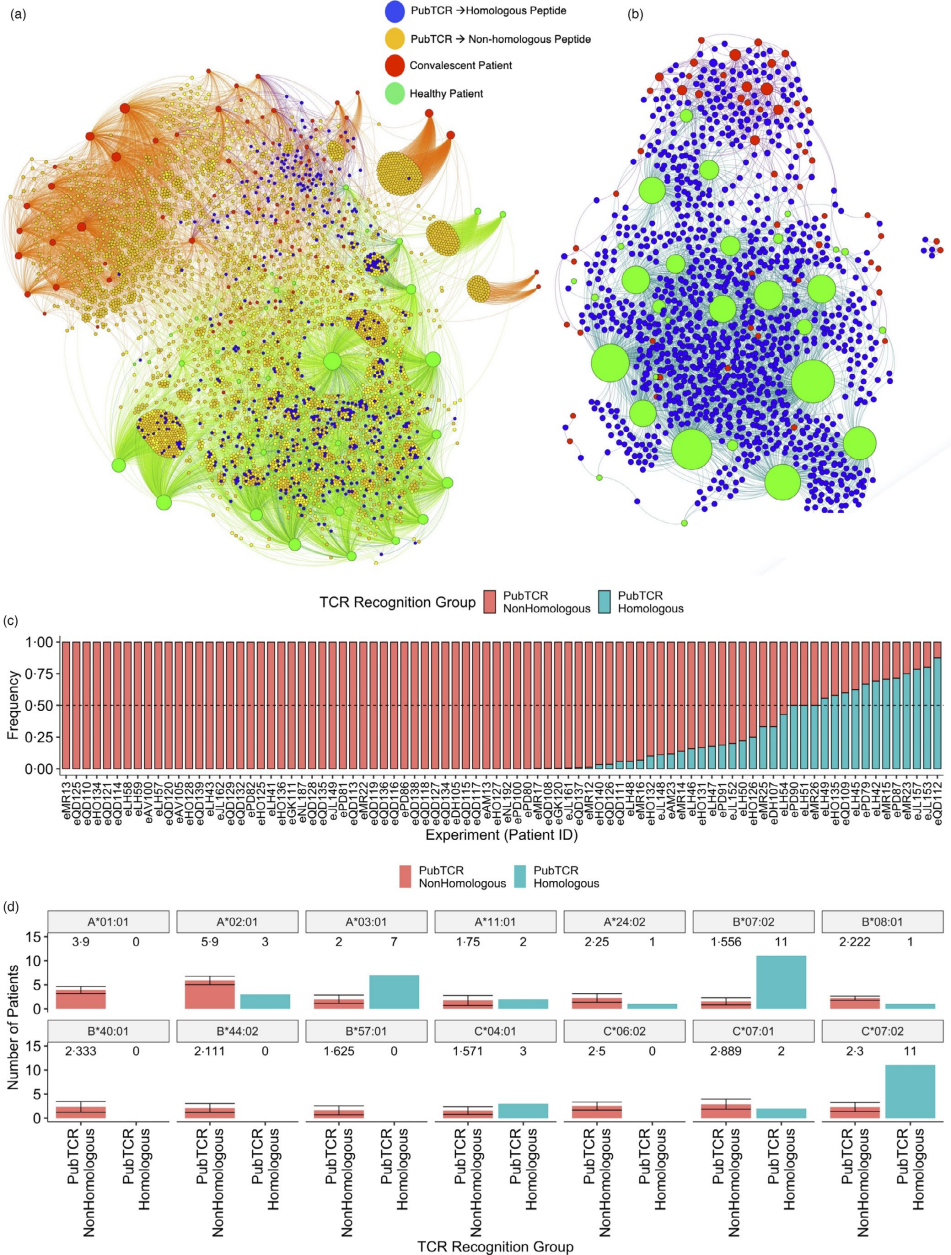
A landscape of T cell responses against SARS‐CoV‐2‐HCoV Homologous and SARS‐CoV‐2‐non‐homologous peptides in healthy or COVID‐19 convalescent individuals: (a) A bipartite network graph showing SARS‐CoV‐2‐specific public TCRs which recognize homologous or nonhomologous SARS‐CoV‐2 peptides in healthy or convalescent patients. TCRs are colour‐coded by whether they recognize only homologous peptides (blue), only non‐homologous peptides (orange) or both (yellow). COVID‐19 convalescent patients are labelled red while healthy controls are labelled green. Node size reflects degree of connectivity, i.e., the quantity of an individual's TCRs which are shared with other patients. (b) A bipartite network graph showing SARS‐CoV‐2 public TCRs that recognize SARS‐CoV‐2‐HCoV homologous peptides. Patient node size reflects the quantity of their TCRs which are shared with another patient. Healthy patients are labelled green, COVID‐19 convalescent are labelled red, and (public) TCRs are labelled blue. (c) A barplot showing the frequency that each convalescent patient's public TCRs recognize SARS‐CoV‐2‐non‐homologous (red) or SARS‐CoV‐2‐HCoV‐homologous (blue) peptides. Patients with identical frequencies are ordered by the number of TCRs. (d) Barplots showing the quantities of COVID‐19 convalescent patients who carry 14 class I HLA alleles of interest. Patients are grouped by whether their public TCRs predominately recognize “non‐homologous” (PubTCR_NonHomologous, *n* = 12, sampled 10 times) or “Homologous” (PubTCR_Homologous, *n* = 12) peptides. For the PubTCR‐NonHomologous group, 12 patients were sampled 10 times and the number of patients carrying alleles was measured. The mean number of patients carrying each allele and the error are visualized. For the PubTCR‐Homologous group, the data contain only 12 patients of interest, thus the number of patients carrying each allele is measured and visualised

Next, using the MIRA dataset we set out to elucidate the landscape of public TCRs in HC and COVID‐19 convalescent patients. We, therefore, generated a bipartite graph comprising all public TCRs cognate for homologous and non‐homologous SARS‐CoV‐2 peptides (Figure 5a, Data File S4). This graph revealed two clear hubs. In the first (green nodes), we observed that healthy subjects were connected to public TCRs which recognize both SARS‐CoV‐2‐HCoV and SARS‐CoV‐2‐non‐homologous peptides. In the second hub (red nodes) comprising convalescent patients, we observed that generally their public TCR repertoires predominately recognize SARS‐CoV‐2‐non‐homologous peptides. Indeed, it appears that cognate TCRs of SARS‐CoV‐2‐HCoV peptides are more pronounced in HC (Figure S7a‐Homologous, Wilcoxon *p* = 0·00029), whereas cognate TCRs of SARS‐CoV‐2‐non‐homologous peptides appear enriched in the convalescent cluster (Figure S7a‐Non‐homologous). Interestingly, we observed a considerable number of TCRs recognising homologous peptides which are common between these two subject clusters, indicating that SARS‐CoV‐2‐HCoV‐specific public TCRs are present not only in COVID‐19 patients but are also expanded from unexposed individuals (Figure 5a,b, Data Files S4 and S5).

Given that in these healthy donors, the TCRs are generally from naïve CD8 ^+^ T cells which are expanded and stimulated with SARS‐CoV‐2 peptide pools and analysed with the ‘MIRA’ assay, the presence of cognate TCRs recognising SARS‐CoV‐2‐HCoV peptides in HC, as well as COVD‐19 patients, may not necessarily translate into pre‐existing T cell immunity. Rather, due to the high similarity between the cognate SARS‐CoV‐2 antigens and (predicted) HCoV presented peptides, we suggest it is plausible that these SARS‐CoV‐2 specific TCRs are cross‐reactive with HCoV peptides. Indeed, consistent with Francis et al. [36] who demonstrate pre‐existing memory CD8 ^+^ T cells to SPR* peptide in 80% of unexposed individuals, we found a set of public TCRs—which are observed in both convalescent and unexposed individuals—recognizing this SARS‐CoV‐2‐HCoV peptide. In this light, we reveal candidate public TCRs and corresponding SARS‐CoV‐2 peptides with high similarity to HCoVs, which should be examined further for cross‐reactive potential.

From these two bipartite graphs, we observed that healthy individuals respond to a balance of SARS‐CoV‐2‐non‐homologous and SARS‐CoV‐2‐HCoV peptides, although it appears that infection *primarily* dictates a dominant recognition of non‐homologous SARS‐CoV‐2 peptides (Figure S7b). For convalescent patients, we observed that public TCR repertoires of the majority (51/86) of patients are almost entirely (≥99%) occupied by TCRs recognizing non‐homologous SARS‐CoV‐2 peptides (Figure 5c). However, in a subset of convalescent patients, public TCRs recognizing SARS‐CoV‐2‐HCoV peptides comprise a substantial fraction of the public repertoire. In fact, for 12 convalescent patients, >50% of their public TCRs recognize SARS‐CoV‐2‐HCoV peptides.

Comparing these two groups of patients, we did not find evidence of a link towards biological sex or age. To explore potential correlates, we first gathered the 12 patients whose public TCRs most dominantly (>50%) recognize SARS‐CoV‐2‐HCoV peptides (labelled *PubTCR*‐*Homologous*), and then via sampling 12 patients 10 times from the set of 51 patients whose public TCRs almost entirely recognize non‐homologous SARS‐CoV‐2 peptides (labelled *PubTCR*‐*Non*‐*homologous*), we compared HLA coding genes of these two groups. We observed that the *PubTCR*‐*Homologous* group is statistically enriched for carrying HLA‐B*07:02, HLA‐C*07:02 and HLA A*03:01, whereas the former group includes a broader set of HLAs among which HLA A*01:01 was more pronounced (Figure 5d). The enrichment of HLA‐B*07:02 in the *PubTCR*‐*Homologous* group is consistent with recent work from Francis et al [36], and these data are in agreement with their claim that CD8 ^+^ T Cell HCoV‐SARS‐CoV‐2 cross‐reactivity may be conditioned by HLA.

Employing these two groups and sampling a set of healthy patients (*n* = 12), we reveal the set of epitopes only recognized by public TCRs in these healthy patients, and those shared with the convalescent *PubTCR*‐*Homologous* group (Data File S6 and S7). Additionally, we reveal peptides only observed in the *PubTCR*‐*Non*‐*homologous* convalescent group, adding to previous insights that SARS‐CoV‐2 infection can provoke T cell responses to a novel set of peptides compared to those expanded from unexposed patients [28].

Recent work shows cross‐reactive *private* TCRs from unexposed subject repertoires, capable of recognising both the SARS‐CoV‐2 SPR* peptide and its LPR* homolog from HCoVs OC43 and HKU1. By mapping out which SARS‐CoV‐2 peptides are recognized in which individuals by private TCRs, we observed SPR* but also an additional set of SARS‐CoV‐2‐HCoV peptides recognized in both healthy and convalescent patients (Figure S7c, Data Files S8 and S9). Lineburg et al., [64] recently reported private TCRs in HLA‐B*07:02 ^+^ unexposed individuals which cross‐react with both the SARS‐CoV‐2 SPR* peptide and the OC43/HKU1 homolog LPR*, which indicates a level of pre‐existing immunity. Of these TCRs, we found two (defined as CDR3b, TRBV, TRBJ) which appear in two HLA‐B*07:02 ^+^ unexposed individuals within the MIRA dataset (Table S2). As these TCRs are now observed in two separate datasets, we, therefore, propose these as public TCRs, capable—as identified by Lineburg et al.,—of cross‐reacting with both SARS‐CoV‐2 SPR* and OC43/HKU1 LPR* peptides.

Taken together, we report the existence of a set of CD8 ^+^ TCRs in both HC and COVID‐19 convalescent patients that recognize SARS‐CoV‐2 peptides with high sequence similarity to a pool of predicted HCoV pMHC. This high sequence similarity indicates the cross‐reactive potential of these TCRs. Primarily, however, we observed that COVID‐19 patients develop public TCR responses to non‐homologous SARS‐CoV‐2 peptides—many of which are not observed in unexposed individuals—indicating that any cross‐reactive potential is limited. For the subset of COVID‐19 patients whose public TCRs are primarily directed towards SARS‐CoV‐2‐HCoV peptides—and are observed in HC—we found distinct HLA profiles. Therefore, in agreement with recent data from Francis et al., we suggest that CD8 ^+^ T cell HCoV‐SARS‐CoV‐2 cross‐reactive potential is apparent, although likely conditioned by patient HLA genotype. It is plausible that these patients may exhibit more robust protection against SARS‐CoV‐2 and its variants.

### Potential conserved coronavirus CD8 ^+^ T cell targets with broad population coverage

Given the emergence of new SARS‐CoV‐2 variants and concern over the theoretical capacity of future mutants to evade current vaccine strategies [1], conserved CD8 ^+^ T cell targets across multiple coronavirus strains with the potential to elicit T cell responses in a large percentage of global populations are of interest. We, therefore, searched our peptide map for SARS‐CoV‐2 peptides with ‘high‐similarity’ matches to *multiple* HCoVs, and with cognate TCRs in the MIRA dataset. To select only the top ‘high‐similarity’ SARS‐CoV‐2‐HCoV matches for this analysis, we applied a more stringent sequence homology threshold. Indeed, in addition to the ‘MatchScore’ and peptide presentation criteria outlined previously (see Methods: Discriminating homologous and non‐homologous SARS‐CoV‐2 peptides), we only retained matches with at least 70% sequence conservation (i.e. allowing 30% amino acid substitution).

**FIGURE 6:**
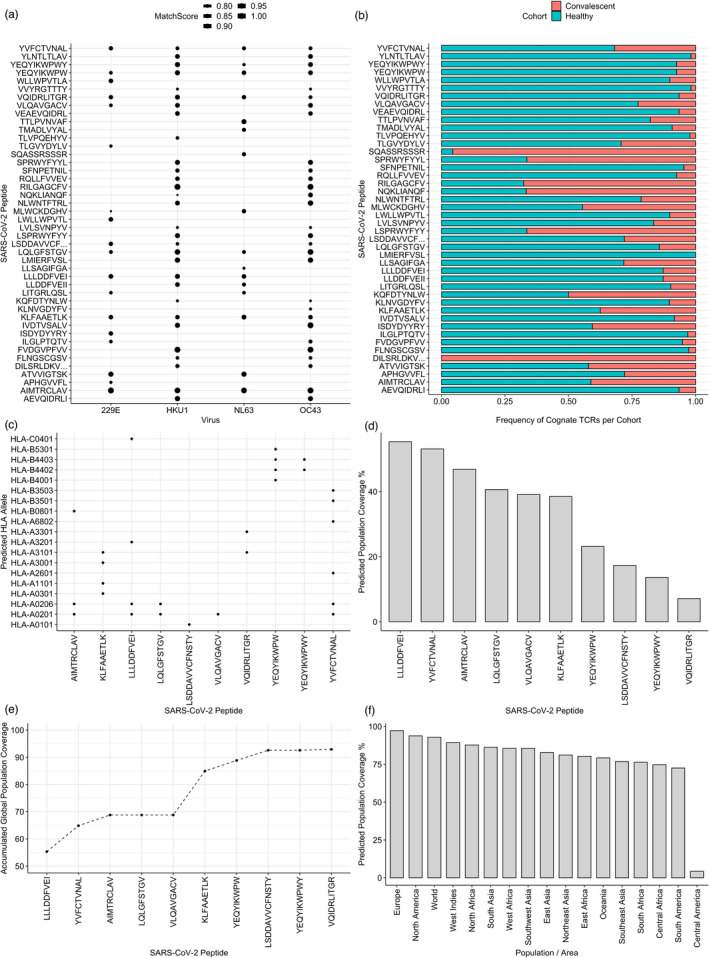
SARS‐CoV‐2 T cell epitopes with known cognate TCRs which are conserved across multiple coronaviruses exhibit broad population coverage: (a) A dot plot showing SARS‐CoV‐2 peptides with high similarity to more than one HCoV that are recognized by TCRs in the MIRA dataset. Size of the dot represents the MatchScore. (b) The frequency of cognate TCRs which recognize these peptides from the COVID‐19 convalescent or healthy cohorts. (c) The HLA alleles predicted to present SARS‐CoV‐2 peptides with high similarity matches to 3 or 4 HCoV strains. (d) Global population coverage as calculated by the ‘IEDB population coverage tool’ for each individual SARS‐CoV‐2 peptide with high similarity matches to 3 or 4 HCoV strains. (e) Accumulated global population coverage predicted by the IEDB population coverage tool. (f) Regional population coverage for the entire set of 10 SARS‐CoV‐2 peptides with matches to 3 or 4 HCoV

We found 44 peptides that match these criteria, 43 of which are recognized by TCRs in both convalescent and HC (Figure 6a,b). We next focused on SARS‐CoV‐2 peptides with high similarity matches in >=3 HCoV strains (Table 2, Data File S10). Of these SARS‐CoV‐2‐HCoV matches, the number of amino acid substitutions ranged between 0 and 3, with a mean of 1·79 and a standard deviation of 0·78. Additionally, while each of these peptides exhibited a high similarity match to either MERS or SARS‐CoV, the majority exhibited homology with both of these viruses (Figure S8a). As well as high conservation across many coronavirus strains, collectively these SARS‐CoV‐2 peptides are predicted to bind multiple HLA alleles (Figure 6c), raising the possibility that this set of peptides may elicit T cell responses in a substantial proportion of the global population.

**TABLE 2:** Highly conserved CD8^+^ T cell peptides across SARS‐CoV‐2 and HCoV strains, with high population coverage

SARS‐CoV‐2 peptide	Virus	Protein	MatchScore
AIMTRCLAV	229E, OC43, HKU1, NL63, SARS‐CoV, MERS_CoV	Replicase polyprotein 1ab, ORF1ab polyprotein, ORF1ab polyprotein, replicase polyprotein 1ab, ORF1ab polyprotein, 1AB polyprotein	1, 1, 0·977, 0·977, 1, 0·977
KLFAAETLK	NL63, 229E, HKU1, OC43, SARS‐CoV, MERS_CoV	Replicase polyprotein 1ab, replicase polyprotein 1ab, ORF1ab polyprotein, ORF1ab polyprotein, ORF1ab polyprotein, 1AB polyprotein	0·881, 0·857, 0·847, 0·847, 1, 0·929
LLLDDFVEI	HKU1, 229E, NL63, OC43, SARS‐CoV	ORF1ab polyprotein, replicase polyprotein 1ab,replicase polyprotein 1ab, ORF1ab polyprotein, ORF1ab polyprotein	0·894, 0·871, 0·86, 0·777, 1
LQLGFSTGV	OC43, HKU1, 229E, NL63, MERS_CoV, SARS‐CoV	ORF1ab polyprotein, ORF1ab polyprotein, replicase polyprotein 1ab, replicase polyprotein 1ab, 1AB polyprotein, ORF1ab polyprotein	0·977, 0·955, 0·809, 0·809, 1, 1
LSDDAVVCFNSTY	229E, HKU1, OC43, SARS‐CoV, MERS_CoV	Replicase polyprotein 1ab,ORF1ab polyprotein, ORF1ab polyprotein, ORF1ab polyprotein, 1AB polyprotein	0·843, 0·789, 0·789, 0·872, 0·789
VLQAVGACV	HKU1, OC43, 229E, NL63, SARS‐CoV, MERS_CoV	ORF1ab polyprotein,ORF1ab polyprotein, replicase polyprotein 1ab, replicase polyprotein 1ab, ORF1ab polyprotein, 1AB polyprotein	0·876, 0·876, 0·795, 0·773, 1, 0·832
VQIDRLITGR	HKU1, 229E, NL63, OC43, SARS‐CoV, MERS_CoV	Surface glycoprotein (all)	0·887, 0·845, 0·845, 0·804, 1, 0·804
YEQYIKWPW	HKU1, OC43, NL63, 229E, SARS‐CoV	Surface glycoprotein (all)	0·903, 0·873, 0·855, 0·794, 1
YEQYIKWPWY	HKU1, OC43, NL63, SARS‐CoV	Surface glyco protein (all)	0·913, 0·886, 0·775, 1
YVFCTVNAL	229E, NL63, HKU1, OC43, SARS‐CoV	Replicase polyprotein 1ab, replicase polyprotein 1ab, ORF1ab polyprotein, ORF1ab polyprotein, ORF1ab polyprotein	0·84, 0·818, 0·809, 0·809, 1

We next sought to determine the extent in global and regional populations that these CD8 ^+^ T cell targets may elicit T cell responses individually and accumulatively. We, therefore, used the IEDB population coverage tool [65], which employs global HLA allele prevalence data to predict the percentage of individuals in a regional population to respond to a given epitope set. Starting with each SARS‐CoV‐2 peptide and predicted HLAs individually, we find considerable coverage of 55·32% for ‘LLLD*’, while ‘VQID*’ exhibits the lowest predicted coverage of 7·09% (Figure 6d).

Similarly to a previous approach by Ahmedid et al [66], we set out to predict the accumulated global population coverage of the set. We found that 8 peptides collectively produce >90% global coverage, while the entire set is predicted to elicit T cell responses in 92·93% of the global population (Figure 6e). Regionally, Europe and North America exhibited the highest predicted coverage (Figure 6f). Of note, Africa and Asia also exhibited high predicted coverage. Central America (defined as Guatemala and Costa Rica) exhibited low coverage of 7%. It is unclear why, and further investigation is necessary to produce a peptide set with high coverage in these countries.

Overall, we identified a set of 10 SARS‐CoV‐2 immunogenic peptides, each highly conserved across coronavirus strains, which collectively provide global population coverage of ~93%. We believe that this is an encouraging insight in the search for pan‐coronavirus T cell targets, and additionally propose these as top candidates for cross‐protective immunity.

## DISCUSSION

Our work demonstrates that T cells specific to SARS‐CoV‐2 peptides with high similarity to HCoV predicted pMHC can be expanded from naïve individuals and that these cognate public TCRs are also observed in a subset of recovered COVID‐19 patients. This finding firstly suggests that SARS‐CoV‐2‐unexposed individuals could mount T cell responses to HCoVs that—due to peptide similarity ‐ could be cross‐reactive with SARS‐CoV‐2 antigens. Furthermore, we propose that while COVID‐19 disease appears to primarily direct responses against non‐homologous SARS‐CoV‐2 peptides, patients with certain HLA alleles (e.g. HLA‐B*07:02, ‐C*07:02, ‐A*03:01) may be more likely to possess SARS‐CoV‐2‐HCoV cross‐reactive CD8 ^+^ T cells. It is, therefore, plausible that SARS‐CoV‐2 naïve individuals with certain HLAs may be at lower risk of severe disease—or experience augmented vaccine responses—if previously exposed to endemic coronaviruses, however, a direct link to pre‐existing immunity requires further investigation.

Indeed, our analysis indicates that after SARS‐CoV‐2 infection, a subset of individuals has memory T cells that primarily recognize SARS‐CoV‐2‐HCoV peptides. In these convalescent patients, it is unclear whether infection itself and/or prior exposure to HCoVs are driving this subset of individuals to select for these peptides. There is conflicting evidence surrounding the existence of memory SARS‐CoV‐2 cross‐reactive CD8 ^+^ T cells in unexposed individuals [36, 37, 64], and a limitation of our work is that we could not provide a direct link to pre‐existing immunity, because from healthy donors the MIRA dataset only evaluated expanded naïve T cells and did not examine anti‐viral efficacy of the responding T cells. Indeed, although we cannot determine the cause or timeframe of this selection of SARS‐CoV‐2‐HCoV peptides in this subset of individuals, the potential implications are interesting. It is plausible that these patients may exhibit more robust protection against SARS‐CoV‐2 variants, HCoVs or even future emerging coronavirus strains. Future work should explore any immunity benefit of infection‐induced cross‐reactive T cell responses, and in addition, it will be interesting to examine whether vaccination against SARS‐CoV‐2 can induce T cell memory that is cross‐reactive with SARS‐CoV‐2 variants and/or wider coronaviruses in such individuals. Furthermore, by our identification of a set of 10 potentially cross‐reactive peptides with broad population coverage, it is possible that these peptides could be employed to test which patients exhibit cross‐reactive phenotypes e.g. after vaccination with relevant antigens.

More broadly, data are beginning to demonstrate distinct vaccine‐induced responses linked to differential patient exposure to SARS‐CoV‐2 [3, 4]. In turn, it is possible that COVID‐19 vaccine boosted cross‐reactive immune responses may influence vaccine‐induced protection [28]. Indeed, it will be important to explore whether COVID‐19 vaccination can boost any infection‐induced cross‐reactive T cell memory and whether this affects the robustness of protection from SARS‐CoV‐2 variants or wider coronaviruses.

SARS‐CoV‐2 reactive CD8 ^+^ T cells have been associated with milder disease [67], and as previously mentioned, conflicting evidence has recently emerged regarding the presence of pre‐existing CD8 ^+^ T cells in unexposed patients. Nguyen et al [37], found that SARS‐CoV‐2‐specific CD8 ^+^ T cells in Australian pre‐pandemic samples, including those recognising the immunodominant HLA‐B*07:02‐SPR* complex, predominately displayed a naïve phenotype, indicating a lack of pre‐existing memory conferred by HCoV. In contrast, Francis et al [36], found that ~80% of unexposed individuals carrying HLA‐B*07:02 show a pre‐existing CD8 ^+^ T cell response to HLA‐B*07:02‐SPR*. Francis et al argue that these pre‐existing memory pools are likely induced by prior exposure to HCoV, and that only a subpopulation of individuals carrying specific HLA would possess such memory T cells. Our work is consistent with a subset of COVID patients enriched for carrying HLA‐B*07:02, and we observed that in these patients, their public T cells respond primarily to SARS‐CoV‐2‐HCoV peptides. Despite not providing a link to memory vs naïve responses, we build upon existing work by proposing additional alleles which may be carried by individuals who possess cross‐reactive T cells, as well as those which appear depleted or absent in these individuals. Few studies have examined associations between HLA type and COVID disease or its severity [36, 68, 69]. Nevertheless, the emerging picture is indicating that HCoV‐SARS‐CoV‐2 cross‐reactivity is conditioned by multiple factors including HLA genotype. Together, we provide a landscape of TCR‐pMHC interactions (all TCR‐pMHC interactions used in the analyses are found in Data File S11) which may be involved in HCoV‐SARS‐CoV‐2 cross‐reactivity and provide a framework for further anti‐viral mechanistic studies.

Although our study provides a map of homologous and non‐homologous SARS‐CoV‐2 peptides to date and offers the extent to which one may expect CD8 ^+^ T cells cross‐reactivity between HCoVs and SARS‐CoV‐2, a limitation is that for cross‐reactivity insights, we had to limit ourselves only on CD8 ^+^ T cells for which both peptides and their cognate TCRs information were available. Additionally, our approach for identifying homologous sequences seems to work better for MHC class I peptides that are considerably shorter in length than their class II counterparts. With a more suitable metric for longer peptides, one may substantiate our insights for class II.

Our metric for discriminating homologous and non‐homologous peptides is based on three factors: (1) sequence homology at 50%, (2) physicochemical similarity of 75% and (3) both source and target peptides must be presented by the same HLA. Of these three, 50% of sequence homology may seem too relaxed. In support of our use of this threshold we note that: (a) factors 2 and 3 are additionally applied to compensate for this, (b) we have checked our results with 70% sequence homology and observed that main conclusions are robust, (c) as this map is suggested for further functional validation, we favour minimizing false negatives at the cost of potential false positives.

Through examining the potential for cross‐reactivity between SARS‐CoV‐2 and HCoV strains, we have predicted that a set of 10 highly conserved immunogenic peptides could mount CD8 ^+^ T cell responses in >90% of the global population. These peptides have been reported previously in *in silico* and experimental work [26, 70, 71, 72, 73] however to our knowledge their large accumulated global population coverage has not yet been reported. Some of these peptides exhibit similar population coverage although with different HLA profiles, therefore it may be possible to tailor a smaller set of peptides to specific regions of interest (based on local HLA frequency), thus maximising coverage with a minimal set of peptides. A very recent study [74] has shown in healthcare workers with repeated exposure to SARS‐CoV‐2, a proportion of these individuals who did not develop symptomatic disease had pre‐existing T cells which targeted ORF1ab (NSP7/12/13) epitopes with similarity to HCoVs. Given that these pre‐existing T cells target a highly conserved region of SARS‐CoV‐2 (and other coronaviruses), Swadling et al speculate that vaccines that boost such T cells may lead to long‐lasting protection against SARS‐CoV‐2 and wider coronaviruses, complementing the current spike‐focused vaccines. Consistent with these insights, 7/10 of the highly conserved epitopes identified in the current study with predicted high population coverage are from ORF1ab, although further analysis is required to determine the extent these epitopes may be recognized by cross‐reactive T cells. Our work firstly identifies these peptides as top candidates for cross‐reactivity. Second, we propose that their high conservation across strains may be of interest as pan‐coronavirus targets, to assist ongoing work in search of mitigation strategies to reduce the threat from mutant variants of emerging coronaviruses [75, 76, 77].

A complex facet of severe COVID‐19 disease and its diverse clinical manifestation is immunopathogenesis. Indeed, exacerbated immune responses including cytokine storm are a primary clinical characteristic in severe COVID‐19 patients. Aberrant transcriptional programming has been observed in response to SARS‐CoV‐2 [78], characterized by a failure of type‐1 and ‐3 interferon responses and simultaneous high induction of chemoattractants. While the growing evidence for pre‐existing HCoV cross‐reactive memory T cell responses may simply translate into an immunity benefit in some patients, in concert with data from MERS and SARS‐CoV‐1, there is considerable evidence that cross‐reactive T and B cell responses may, on the contrary, be involved in immunopathology with SARS‐CoV‐2.

Venkatakrishnan et al. [79], identified peptides that are identical between SARS‐CoV‐2 and the human proteome. Their work demonstrates that the genes giving rise to these peptides are expressed in tissues implicated in COVID‐19 pathogenesis. Our work expands their insights, by identifying SARS‐CoV‐2 peptides that are experimentally confirmed to be immunogenic, with high similarity to the human proteome. Consistent with their conclusions, we find similarities of immunogenic SARS‐CoV‐2 peptides to human genes e.g. CCL3, CCL31 and CD163. These insights are of particular interest given the elevated cytokine and chemokine responses in severe COVID patients.

While the negative thymic selection is effective in deleting T cell precursors with high avidity for self pMHC, some autoreactive T cells can escape negative selection [80]. In such cases, an array of peripheral tolerance mechanisms play a prominent role in regulating responses in healthy tissues. Such mechanisms range from, the exclusion of naïve T cells from nonlymphoid peripheral tissues, reducing the likelihood of contacting a tissue‐resident APC expressing self‐antigen [80, 81], to anergy where T cells do respond to self‐peptide can be eliminated or inactivated [82]. Nevertheless, there is evidence that viral antigens that are structurally similar to self‐antigens can be involved in inducing autoimmunity via molecular mimicry [29]. Additionally, it is hypothesized that non‐specific antiviral immune responses may lead to the release of self‐antigens by damaged cells, resulting in ‘bystander activation’ of autoreactive T cells [29, 83]. In this light, we propose these immunogenic SARS‐CoV‐2 peptides with high similarity to self, as candidates which may exhibit immunopathological or autoimmune associations.

In conclusion, we have employed an *in*‐*silico* approach to examine the evidence surrounding cross‐reactive SARS‐CoV‐2 CD8 ^+^ T cell responses. We observed a set of SARS‐CoV‐2 candidates with high similarity to the human proteome and suggest investigation into whether they provoke immunopathology. We have also provided evidence of CD8 ^+^ T cell cross‐reactivity, not only to an extent that indicates that naïve individuals could mount cross‐reactive responses to SARS‐CoV‐2 and common‐cold coronaviruses, but we also found that SARS‐CoV‐2 infection induces CD8 ^+^ T cell responses against peptides with high similarity to HCoV in some COVID‐19 patients. We build upon existing evidence that such cross‐reactivity is conditioned by the presence of specific HLA alleles and envision that the insights presented here are leveraged to explore whether these potentially cross‐reactive T cells and cognate pMHCs influence COVID‐19 disease heterogeneity, vaccine‐ or infection‐induced protection from SARS‐CoV‐2 and its emerging variants of concern.

## METHODS

### Data processing and analysis

All data processing and analysis were performed using the R plugin for Pycharm 2020, in either R 40.3 or 4.0.1. Visualisations were made using R library *ggplot*. Any graph clustering (i.e Figure 3c) was performed using the function *daisy* from the library *cluster*.

### Curating a pool of SARS‐CoV‐2 class I and II peptides

Human immunogenic and non‐immunogenic SARS‐CoV‐2 peptide data were gathered from both the IEDB and the Virus Pathogen Resource (VIPR) (accessed 11‐02‐2021). ‘T cell’ assay, ‘Human’ host and SARS‐CoV‐2 organism options were selected. If an observation was found in both datasets, the one from the IEDB was retained. Protein names were cleaned and standardized where possible. Immunogenic peptides not observed in either the IEDB or VIPR were also gathered from the ‘MIRA’ dataset which maps cognate TCRs and SARS‐CoV‐2 peptides.

### Retrieval of coronavirus proteome sequences

NCBI reference genomes were gathered for OC43 (https://www.ncbi.nlm.nih.gov/nuccore/1578871709/), HKU1 (https://www.ncbi.nlm.nih.gov/nuccore/NC_006577.2), 229E (https://www.ncbi.nlm.nih.gov/nuccore/NC_002645.1), NL63 (https://www.ncbi.nlm.nih.gov/nuccore/49169782/) and SARS‐CoV‐2‐Wuhan (https://www.ncbi.nlm.nih.gov/nuccore/nc_045512.2).

### MHC Presentation Prediction

Antigen presentation by MHC class I was predicted using NetMHCpan v4.1 against HLA‐A*0101, 0201, 0301, 2402, HLA‐B*0702, 4001, 0801, and HLA‐C*0702, 0401, 0701 alleles. Antigen presentation by MHC class II was predicted using netMHCIIpan against the most common sets of alleles found in the IEDB, for which this model can make predictions. The alleles are: DRB1‐0101, 0102, 0301, 0401, 0402, 0402, 0404, 0701, 0801, 0901, 1001, 1101, 1104, 1201, 1202, 1301, 1302, 1303, 1401, 1406, 1501, 1502, 1601, 1602, DRB3_0101, 0202 and DRB5_0101, 0102. Peptides with a rank score <=2·0 were classified as binders.

### HLA ligand enrichment analysis for SARS‐CoV‐2 proteins

To provide reasonable statistical inference, we only examined proteins longer than 100 amino acids. To compute enrichment or depletion, we followed the approach by Karnaukhov et al. First, we predicted using netMHCpan v 4.1 the number of ligands *Ni* of length *l* from each SARS‐CoV‐2 protein *i* which adheres to the criteria. The probability of a HLA allele presenting a peptide was computed as the average number of ligands per allele:

p=<Ni>/<Li>

where Li is the corrected protein length (length of protein – *l*), and <:> denotes the average over the assessed SARS‐CoV‐2 proteins. It follows that the probability of observing a given number of ligands from each SARS‐CoV‐2 protein is computed using the binomial distribution as:

$$P\left(\mathrm{Ni}\right)=P_{\mathrm{binom}}\left(\mathrm{Ni}|p,\;\mathrm{Li}\right)$$

The logs odds ratio (enrichment or depletion) is calculated as:

$$\log(\frac{\mathrm{Ni}}{p\mathrm{Li}})$$

### Discriminating Homologous and Non‐homologous SARS‐CoV‐2 Peptides

To compare a SARS‐CoV‐2 peptide *a*, of length *N* to a proteome of interest, all possible linear peptides of length *N* were generated from said proteome. This can be thought of as scanning along the proteome of interest with a step size of 1, generating all peptides of length *N*. The deriving protein was recorded. Three metrics—which all must be satisfied—were used to determine whether a peptide is considered homologous with HCoV or non‐homologous to SARS‐CoV‐2. We below describe each metric and then explain the three thresholds which all must be achieved for a peptide to be classified as ‘homologous’.

First, once all peptides from the proteome of interest of length *N* are generated, a similarity index we call the ‘MatchScore’ is calculated for each pairwise comparison. This metric is charged with assessing physicochemical similarity between two peptides of interest. For each SARS‐CoV‐2 peptide, the highest ‘MatchScore’ against each HCoV protein is retained and the rest are discarded. To calculate the ‘MatchScore’, we employ the method designed by Bresciani et al [41]. Briefly, for two peptides *a* or *b* of length N, the similarity score is given as:

$$\mathrm{MatchScore}=\frac{\mathrm{bl}(a,b)}{\sqrt{\mathrm{bl}\left(a,a\right)\times \mathrm{bl}\left(b,b\right)}}$$

where bl(*a*,*b*) is the BLOSUM62 score for peptide *a* vs *b*, and bl(*a*,*a*) is the BLOSUM62 score for peptide *a* vs *a*, etc. BLOSUM62 local‐global alignment scores (local or global would produce the same score for a pairwise alignment of lengths N vs N) were computed using the pairwiseAlignment function from the R package Biostrings, with high gap penalties (opening and extension of both 100). The MatchScore function produces a score where 1 reflects an exact match, i.e no mismatches in two sequences, and 0 reflects high dissimilarity.

#### Criteria 1: A homologous peptide and its HCoV match must have a MatchScore of >0·75

The second metric is based on sequence homology between two sequences, essentially reflecting the proportion of amino acid positions in the SARS‐CoV‐2 peptide, which are conserved in the HCoV match. This is calculated as:

$$\mathrm{ProportionMismatched}=\;\frac{\mathrm{HammingDistance}}{\mathrm{Length}}$$

where ‘HammingDistance*’* is the hamming distance between two peptides of interest, which calculates the number of different positions, and ‘Length*’* is the length of the compared peptides.

#### Criteria 2: The *ProportionMismatched* between a homologous peptide and its HCoV match must be <0·5 (50%)

Naturally, the inverse of this is true, in that at least 50% amino acid conservation between a SARS‐CoV‐2 peptide and HCoV match must be observed for the peptide to be considered ‘homologous’.

The third metric is based on the predicted presentation by HLA of the SARS‐CoV‐2 peptide and its HCoV match.

#### Criteria 3: Both the SARS‐CoV‐2 peptide and its HCoV match must be predicted to bind at least one common HLA allele

All three criteria must be satisfied for a SARS‐CoV‐2 peptide to be classified as a homologous peptide and also for a match from HCoV to be considered a homologous match. *doParallel* and *foreach* functions were used to parallelize the processing.

### Sequence logos of SARS‐CoV‐2 Homologous and Non‐homologous peptides

The amino acid usage of SARS‐CoV‐2 homologous/non‐homologous peptides of length 9 were compared using the ggseqlogo function of the library *PepTools*.

### Sequence similarity with the human proteome and human microbiomes

Here, the same similarity criteria were employed as in the previous HCoV section. However, in contrast with HCoV comparison, due to the size of the human proteomes and microbiomes, the best match against the whole proteome is retained. *doParallel* and *foreach* functions were used to parallelize the processing.

The reference human proteome sequence was downloaded in fasta format from UniProt https://www.uniprot.org/proteomes/UP000005640

Human gut and airways microbiome sequences were downloaded from the HMP Data Analysis and Coordination Center http://www.hmpdacc.org/HMRGD. The complete set of genomes was downloaded in fasta format in ‘Protein multifasta (PEP) format’. For gut, the body site was specified as ‘gastrointestinal tract’. 457 and 50 gut and airway microbiota were available respectively.

### Comparing sequence dissimilarity against the human proteome for immunogenic vs nonimmunogenic SARS‐CoV‐2 peptides

The best ‘MatchScore’ for each SARS‐CoV‐2 immunogenic and nonimmunogenic peptide were compared for various peptide lengths. Wilcoxon test was used to assess significance.

### Human gene sets with sequence similarity to SARS‐CoV‐2 immunogenic peptides

The SARS‐CoV‐2 peptides of lengths 9 and 10 with a similarity score to the human proteome in the top 10 percentile were gathered. Only predicted binders (see MHC presentation prediction) were retained. By retaining the ProteinIDs of each match, the proteins where a high similarity match was observed, were examined.

### CD8 ^+^ T cell cross‐reactivity maps using IEDB receptor data

The entire IEDB receptor data for SARS‐CoV‐2 peptides were downloaded. Bipartite graphs were generated using *iGraph* and *Matrix* libraries in R. Bipartite graphs were projected into one‐mode graphs using the *bipartite_projection* function. All graphs were exported from *iGraph* into Cytoscape v3.82 using the R function *createNetworkFromIgraph* from package *RCy3*. From Cytoscape, ‘.graphml’ files were exported and opened with Gephi. Gephi was used to finalize the diagrams and improve visual aesthetics. Either ‘ForceAtlas’ or ‘Fructerman‐Reingold’ templates were used. Gravity and repulsion parameters were altered to improve visual aesthetics.

### CD8 ^+^ T cell CDR3 Kmer Enrichment

R Package *immunarch* [84]was used to compute Kmer (*K* = 5 in this case) statistics for CDR3 sequences and to visualize enrichment. See https://immunarch.com/articles/web_only/v9_kmers.html for full details.

### Gathering clinical and TCR repertoire data for COVID‐19 patients and healthy subjects

The COVID‐19 MIRA dataset (>160k high‐confidence SARS‐CoV‐2‐specific TCRs) was downloaded from https://clients.adaptivebiotech.com/pub/covid‐2020 with corresponding sample metadata. These data contain TCR repertoire data mapped to SARS‐CoV‐2 epitopes from 5 patient cohorts, including COVID convalescent patients and healthy subjects with no known exposure to SARS‐CoV‐2. Only convalescent patients and healthy subjects were used in the analysis due to the low numbers of subjects for other cohorts.

https://clients.adaptivebiotech.com/pub/covid‐2020

### Motifs analysis for MIRA SARS‐CoV‐2‐specific public TCRs which recognize Homologous vs Non‐homologous Peptides

CDR3b sequences were gathered from the MIRA dataset and grouped into those which recognize only SARS‐CoV‐2‐homologous or only SARS‐CoV‐2‐non‐homologous peptides. Rare lengths <7 or >20 amino acids were excluded. To deal with CDR3 length variability, a simple ‘alignment’ was was performed by introducing *n*‐*k_i_* gaps into the centre of each CDR3 sequence, where *n* = 20, the max CDR3 length in the analysis, and *k_i_* is the length of CDR3 sequence *i*. Sequence logo plots using R package *ggseqlogo* were generated using the ‘aligned’ sequences for each group. Shannon entropy was calculated as described previously [85].

### Comparing MIRA SARS‐CoV‐2‐specific public TCRs with those which recognize epitopes from other viruses

CDR3β sequences comprising public TCRs which recognize SARS‐CoV‐2‐homologous peptides were gathered from the MIRA dataset. All human TCRβ sequences were downloaded from VDJdb (accessed 05/11/21), which contain—amongst other information—CDR3β sequences mapped to an epitope, alongside where the peptide originates (in most cases a pathogen). CDR3b sequences from the MIRA dataset which are observed to recognize CMV, Influenza A, HIV‐I, EBV, etc. from the VDJdb dataset were identified. The overlap of these sequences was visualized using the R package *venn*. After dealing with CDR3β length variability (see methods section above: *Motifs analysis for MIRA public TCRs*), motifs for SARS‐CoV‐2‐specific public TCRs which overlap with Influenza A, or CMV, or EBV were visualized using R package *ggseqlogo*.

### Networks of COVID‐19 patient TCRs recognising Homologous and/or Non‐homologous Peptides

A public TCR is defined as a CDR3 sequence and V and J gene which is observed in more than one patient in the MIRA dataset. All graphs were first generated using *iGraph* in R, exported to Cytoscape using the *createNetworkFromIgraph* function in the *RCy3* package. From cytoscape, all graphs were exported as.graphml files and read into Gephi. In Gephi, either ‘ForceAtlas’ and ‘Fruchterman‐Reingold’ templates were used. In all cases, gravity and repulsion parameters were adjusted to improve visual aesthetics. The size of each node reflects the degree of connectivity.

### Enrichment or Depletion of HLAs in COVID‐19 convalescent patient TCR repertoires

The ‘PubTCR_Homologous’ group of patients was curated by counting the number of distinct public TCRs recognising homologous peptides, for each COVID‐19 convalescent patient in the MIRA dataset. The ‘PubTCR_Non‐homologous’ group was curated by counting the number of distinct public TCRs recognising SARS‐CoV‐2‐non‐homologous peptides for each convalescent patient. For the ‘PubTCR‐Homologous’ group, we observed that for 12 patients, >50% of their public TCR repertoires are cognate for homologous peptides. Therefore, for this group, we focused on these 12 patients. We report how many times each HLA allele was observed amongst this set of patients.

For the ‘PubTCR‐Non‐homologous’ group, we observed that the majority (51) patients had public TCR repertoires almost entirely recognising non‐homologous peptides. For this analysis, we sampled 10 patients, 10 times from these 51 patients, and each time count how many times each HLA allele was observed amongst this set of patients. For each HLA allele, we report the mean and standard deviation of the distributions.

### Estimating population coverage of SARS‐CoV‐2 peptides with high conservation to three or more HCoV

We followed the approach by Ahmedid et al.^66^ Population coverage is an estimate of the proportion of individuals in a given population that may mount a T cell response against a peptide. Population coverage is predicted based on HLA alleles for each immunogenic peptide as predicted by netMHCpan 4.1, leading to individual population coverage of a peptide. To predict accumulated coverage, we began with the peptide with the highest individual coverage “FVDG*”, and incrementally added a peptide and predicted accumulated coverage. The population coverage of a set of peptides (i.e accumulated coverage), is defined as the proportion of individuals able to mount a T cell response to at least one peptide in the set. Python code for the IEDB tool to compute the population coverage was downloaded from http://tools.iedb.org/population/download on 24 November 2020.

## CONFLICT OF INTEREST

GO has co‐filed a patent related to T cells response to SARS‐CoV2. Other authors declare no competing interests.

## AUTHOR CONTRIBUTIONS

HK conceived, designed and supervised the project. PRB performed computational analyses with insights from CL and MPP and AA. HK and PRB interpreted the results. AS, GO‐assisted design, interpretation and supervision. ROB, JW commented on the manuscript. HK, AS funded the project. HK, PRB, wrote the manuscript with contributions from CL, MPP and AA, AS and GO.

## Supporting information

Supinfo

## Data Availability

The raw data used in this study derived from public domain as described in the method. Processed data are provided as supplementary files as well as in Github upon publication of the study.
