# Supplementary material for: HLA‐dependent variation in SARS‐CoV‐2 CD8 + T cell cross‐reactivity with human coronaviruses
Source: Immunology. 2022 Mar 7;166(1):78–103. doi: 10.1111/imm.13451 (PMC9111820; doi:10.1111/imm.13451)
Supplement: Supplementary file 1 — Supinfo [file IMM-166-78-s001.zip › imm13451-sup-0001-Supinfo.pdf]

Supplementary

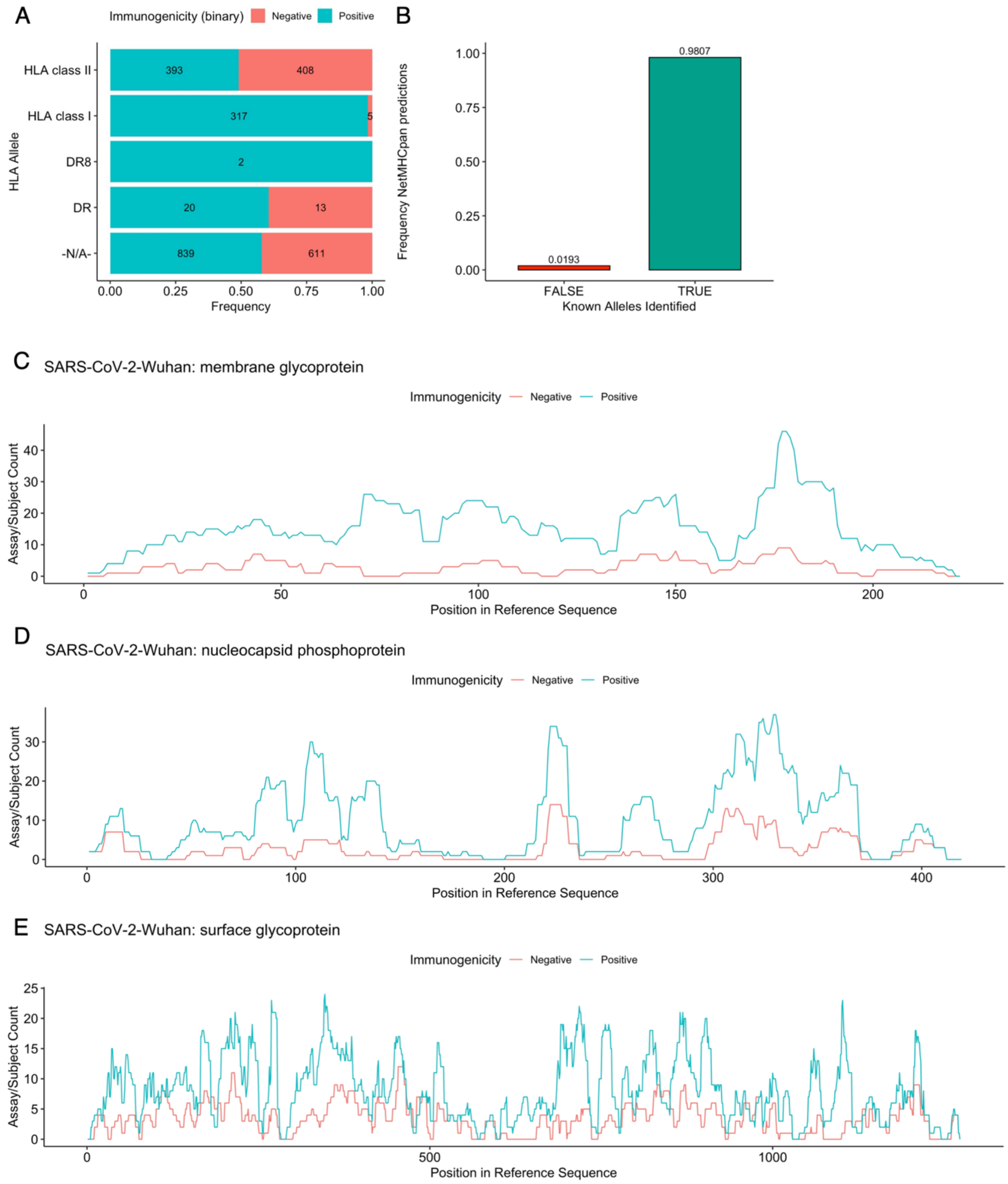

Supplementary Figure 1: A-B) Barplots showing A) the frequency of immunogenic epitopes amongst HLA labels where the specific allele is unknown. B) the frequency of netMHCpan predictions where the correct allele for the HCoV-CoV2 homologous peptide (where known) was identified. C-E) Line plots showing the immunogenic regions of example SARS-CoV-2 proteins from where many immunogenic peptides arise in our dataset, C) membrane glycoprotein, D) nucleocapsid phosphoprotein, E) ‘spike’ surface glycoprotein.

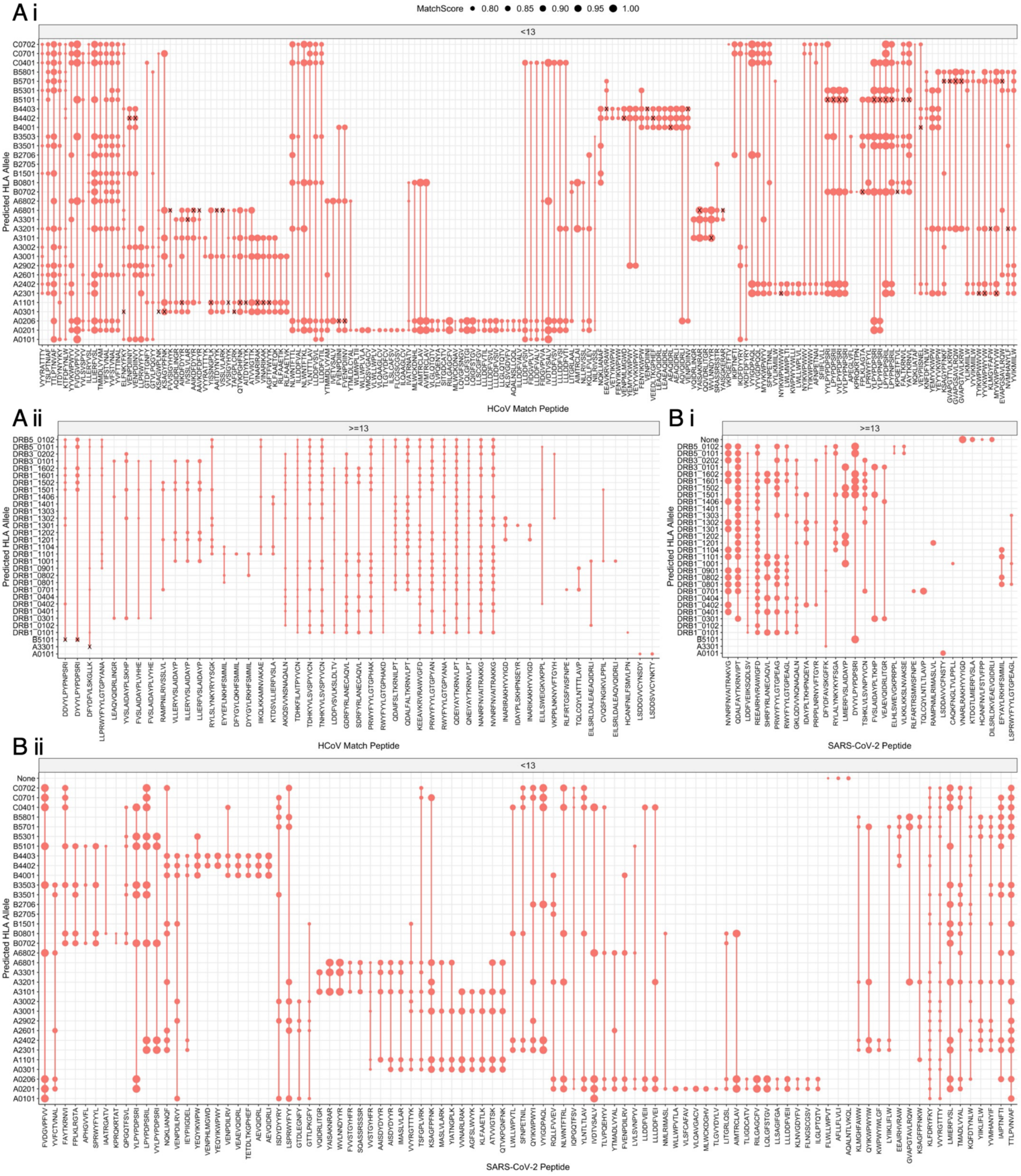

Supplementary Figure 2A-B, continued overleaf.

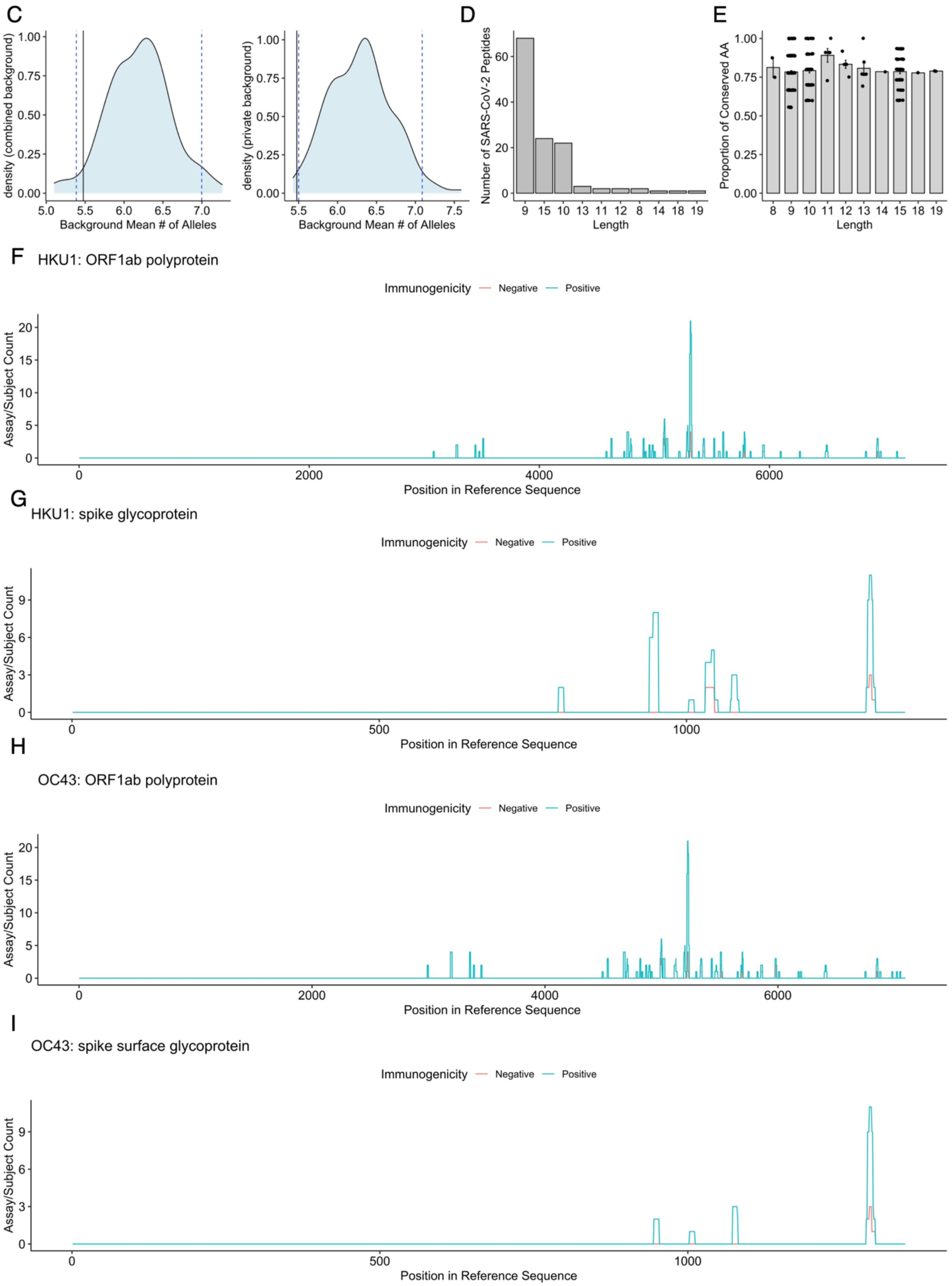

Supplementary Figure 2: A) A bubble chart showing predicted HLA alleles for i) peptides of length <13 and ii) peptides of length >=13, from HCoV with high-similarity to immunogenic SARs-CoV2 peptides. Size of the point reflects the similarity score to the corresponding CoV2 peptide. Lines link multiple predicted alleles. An 'X' indicates – where possible - if the corresponding SARS-CoV-2 peptide counterpart is functionally evaluated as binding the same HLA allele. B) Bubble chart showing predicted HLA alleles for the 126 homologous SARS-CoV-2 immunogenic i) peptides of length <13 and ii) peptides of length >=13. C) Density plots showing the mean number of alleles for which SARS-CoV-2-homologous peptides are predicted to bind (solid black line), compared with a background distribution generated by randomly shuffling the dataset using both non-homologous and homologous peptides (left) or non-homologous peptides only (right). Dashed lines indicate -+ 2 standard deviations from the mean. D) Bar plot showing the distribution of lengths for 126 homologous SARS-CoV-2 immunogenic peptides. E) Bar plot showing the distribution of amino acid conservation between the 285 matches and the 126 SARS-CoV-2 homologous peptides. F-I) Line plots showing the HCoV protein regions which produce immunogenic HCoV-CoV-2 homologous peptides from F) HKU1 ORF1ab polyprotein, G) HKU1 spike glycoprotein, H) OC43 ORF1ab polyprotein, I) OC43 spike glycoprotein.

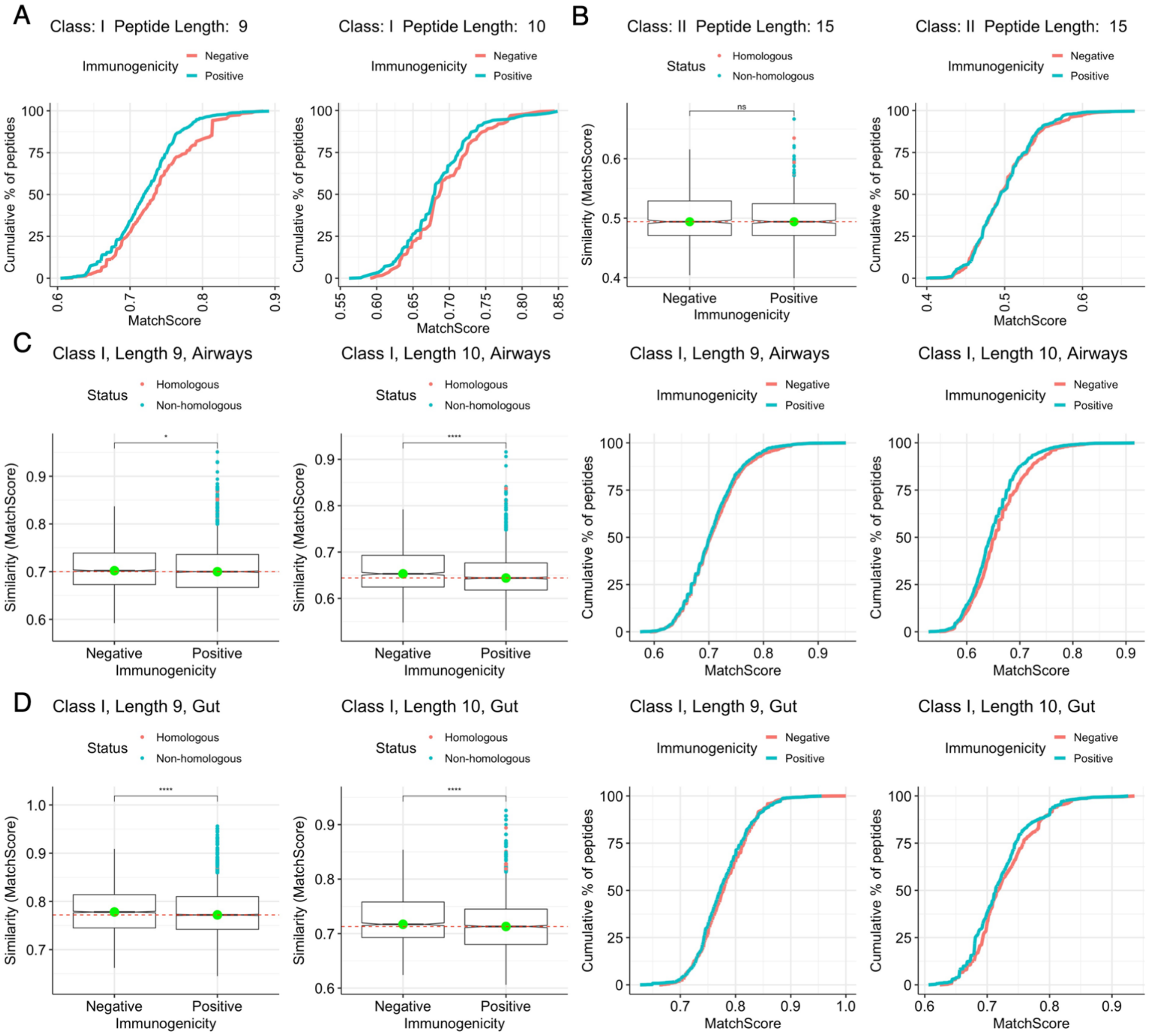

Supplementary Figure 3: A) Empirical cumulative distribution plots for HLA class I peptides of length 9 and 10, showing the cumulative % of peptides to exhibit each MatchScore, color labelled by immunogenicity status. B) A notched boxplot and empirical cumulative distribution plots showing the similarity as evaluated by the MatchScore of nonimmunogenic and immunogenic class II SARS-CoV-2 peptides with sequences derived from the human proteome of length 15 (immunogenic n=955, nonimmunogenic n=953 peptides). C-D) Notched boxplots and empirical cumulative distribution plots showing the similarity of nonimmunogenic and immunogenic SARS-CoV-2 peptides with sequences derived from gut C) and airway microbiomes D).

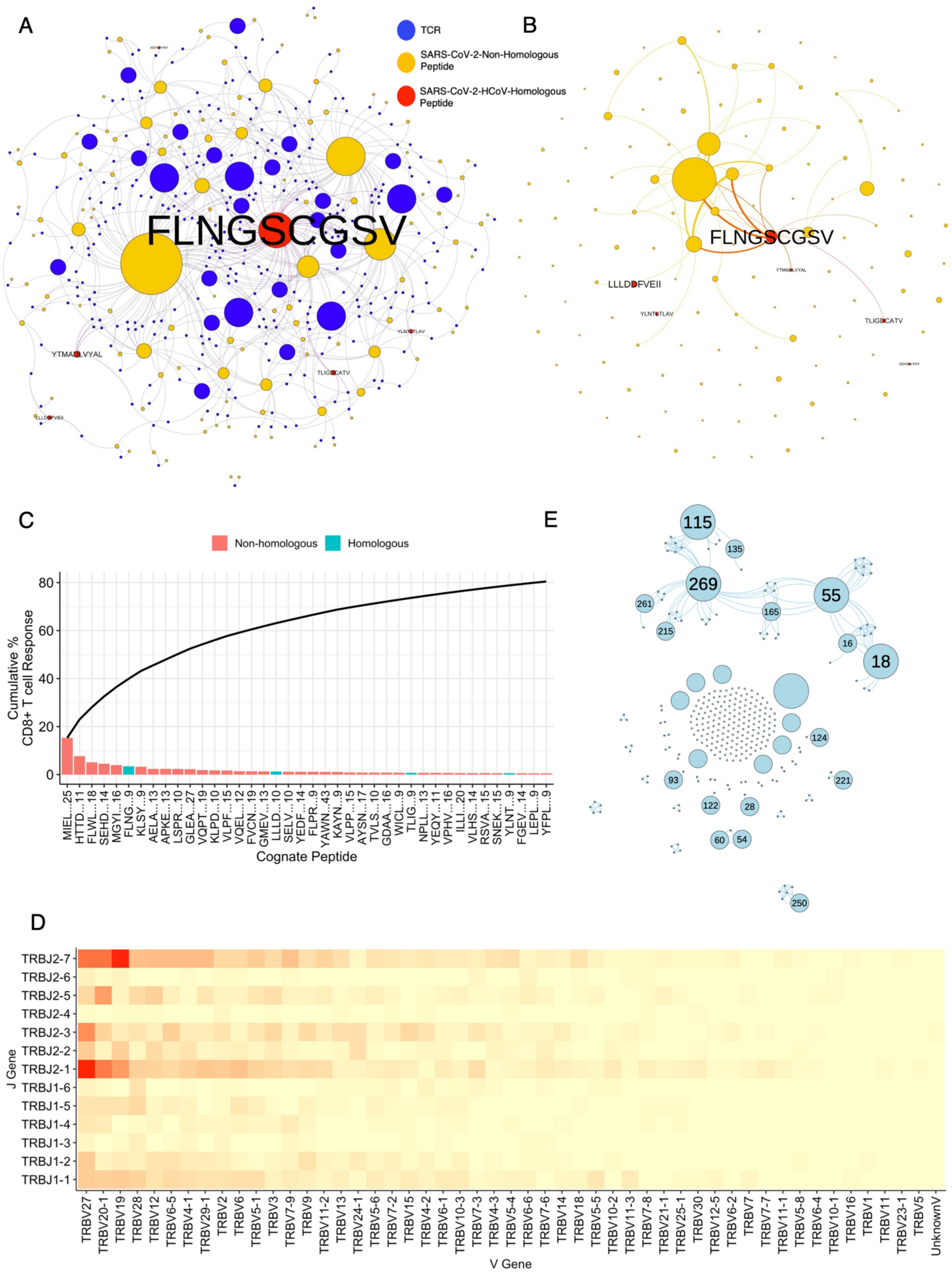

Supplementary Figure 4 A-D, continued overleaf.

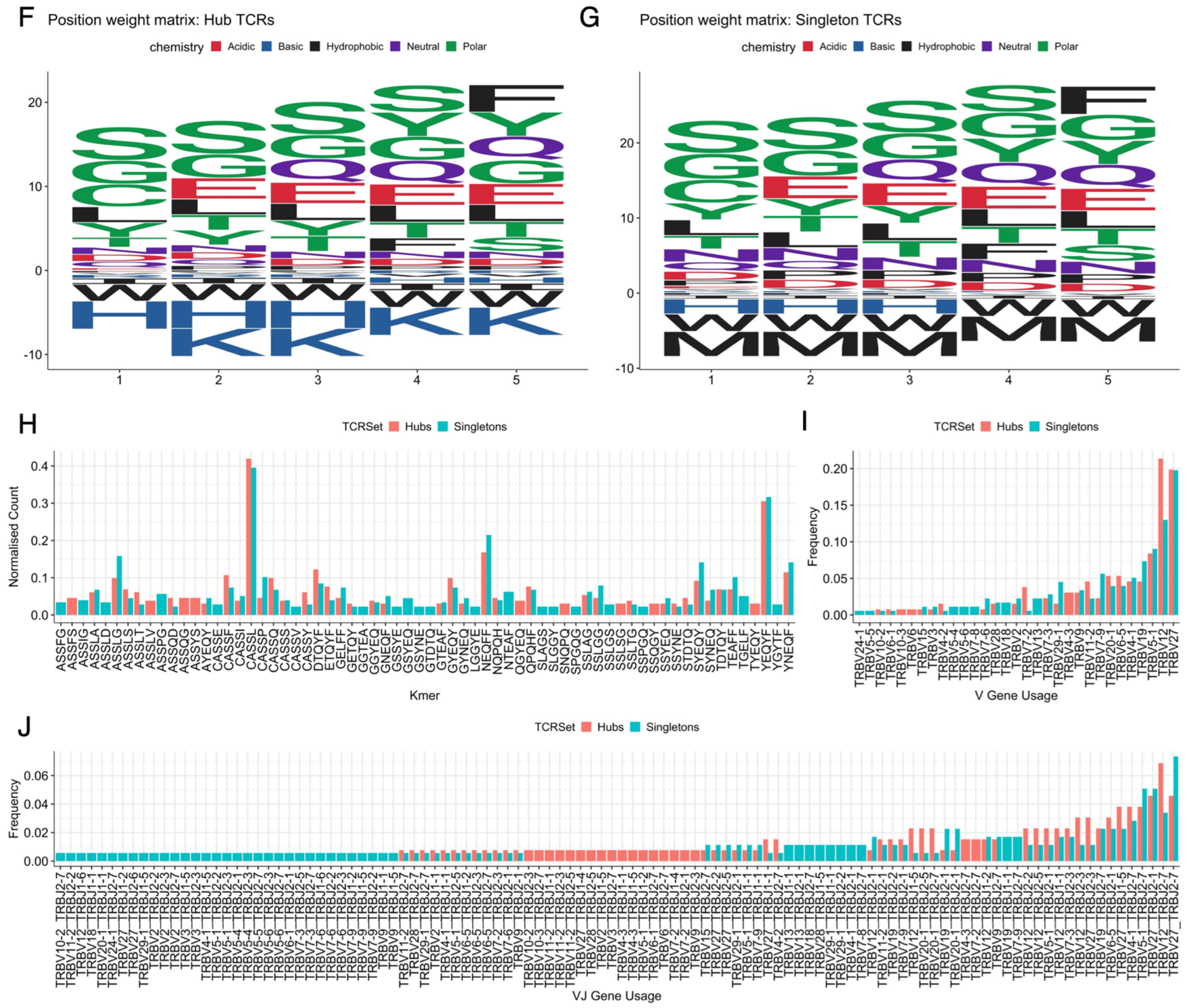

Supplementary Figure 4: SARS-CoV-2 CD8$^+$ T cell cross-reactivity and common-specificity. A) A bipartite network graph depicting the interactions of SARS-CoV-2 immunogenic peptides (SARS-CoV-2-homologous are colored red, -non-homologous are coloured yellow) and their cognate TCRs (blue). Node size represents the degree of connectivity. B) A one-mode network graph illustrating homologous and non-homologous SARS-CoV-2 peptides. An edge between a peptide node demonstrates that a peptide is recognized by the same TCR. Node size reflects the degree of connectivity. C) A barplot showing the top 80% of the cumulative SARS-CoV-2-specific CD8+ T cell response and the peptides recognized, as per the cognate TCR dataset from the IEDB. D) A heatmap showing common V and J gene usage for this 80% of SARS-CoV-2 specific TCRb sequences which recognize the dominant peptides. E) A one-mode network graph showing the common specificity of SARS-CoV-2 specific TCRs. Each node is a TCR, and an edge reflects whether two TCRs recognize the same peptide. Node size reflects the number of peptides recognized by a TCR. F) A sequence logo plot visualizing the position weight matrix for CDR3b 5mers in the IEDB dataset, for “Hub TCRs”, those with considerable common-specificity. G) A sequence logo plot visualizing the position weight matrix for CDR3b 5mers in the IEDB dataset, for “Singleton”, those recognizing only one unique SARS-CoV-2 peptide H) A barplot contrasting the Kmer distribution for“Hub” and “Singleton” TCRs. The count is normalized by the number of TCRs in each group (Hub or Singletons). I) A barplot contrasting the V gene usage of ”Hub” and “Singleton” TCRs. Y axis shows the frequency to which the V gene is used in each group. J) A barplot contrasting the V-J gene usage of ”Hub” and “Singleton” TCRs. Y axis shows the frequency to which the V-J gene combination is used in each group.

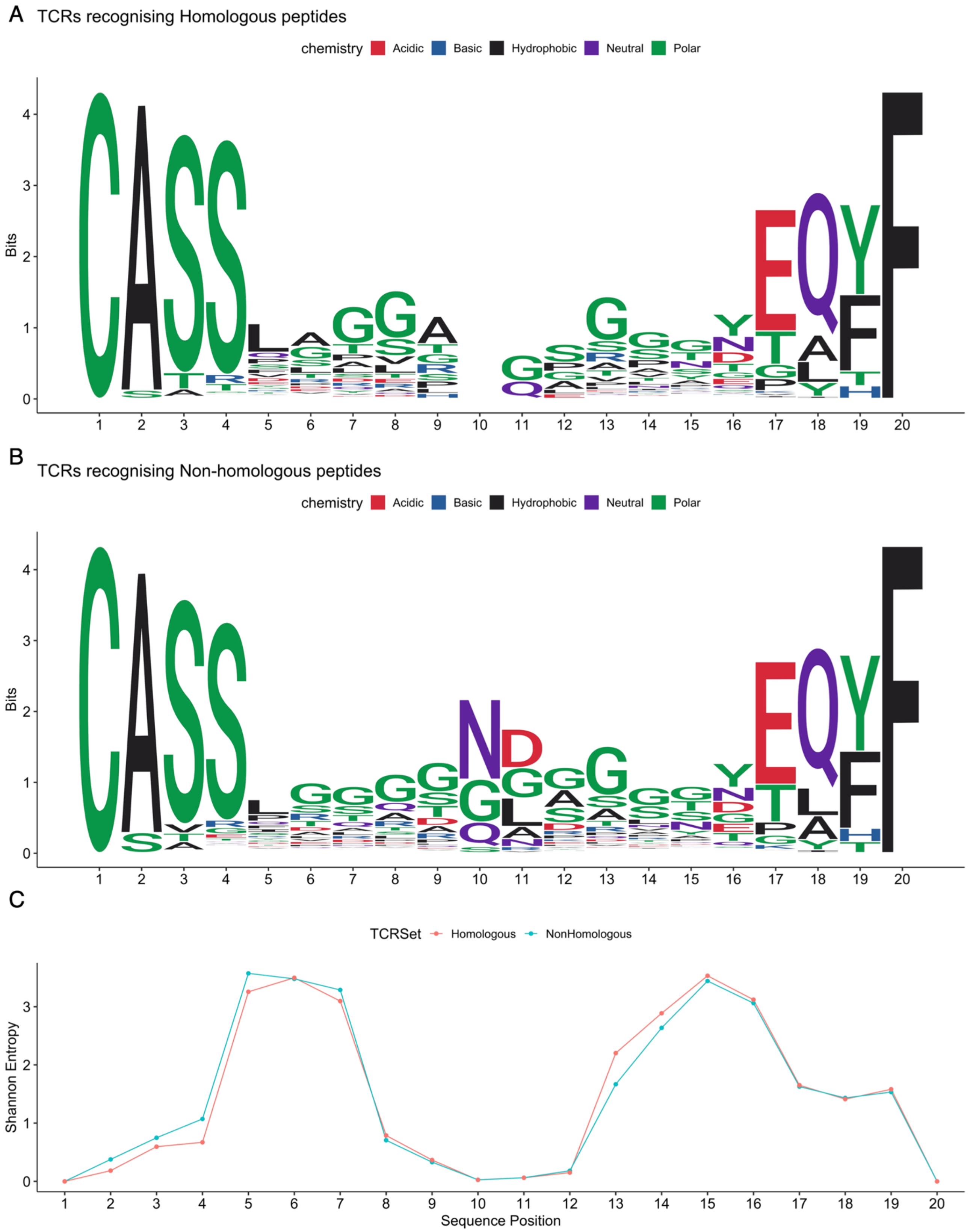

Supplementary Figure 5 A-C: Continued overleaf.

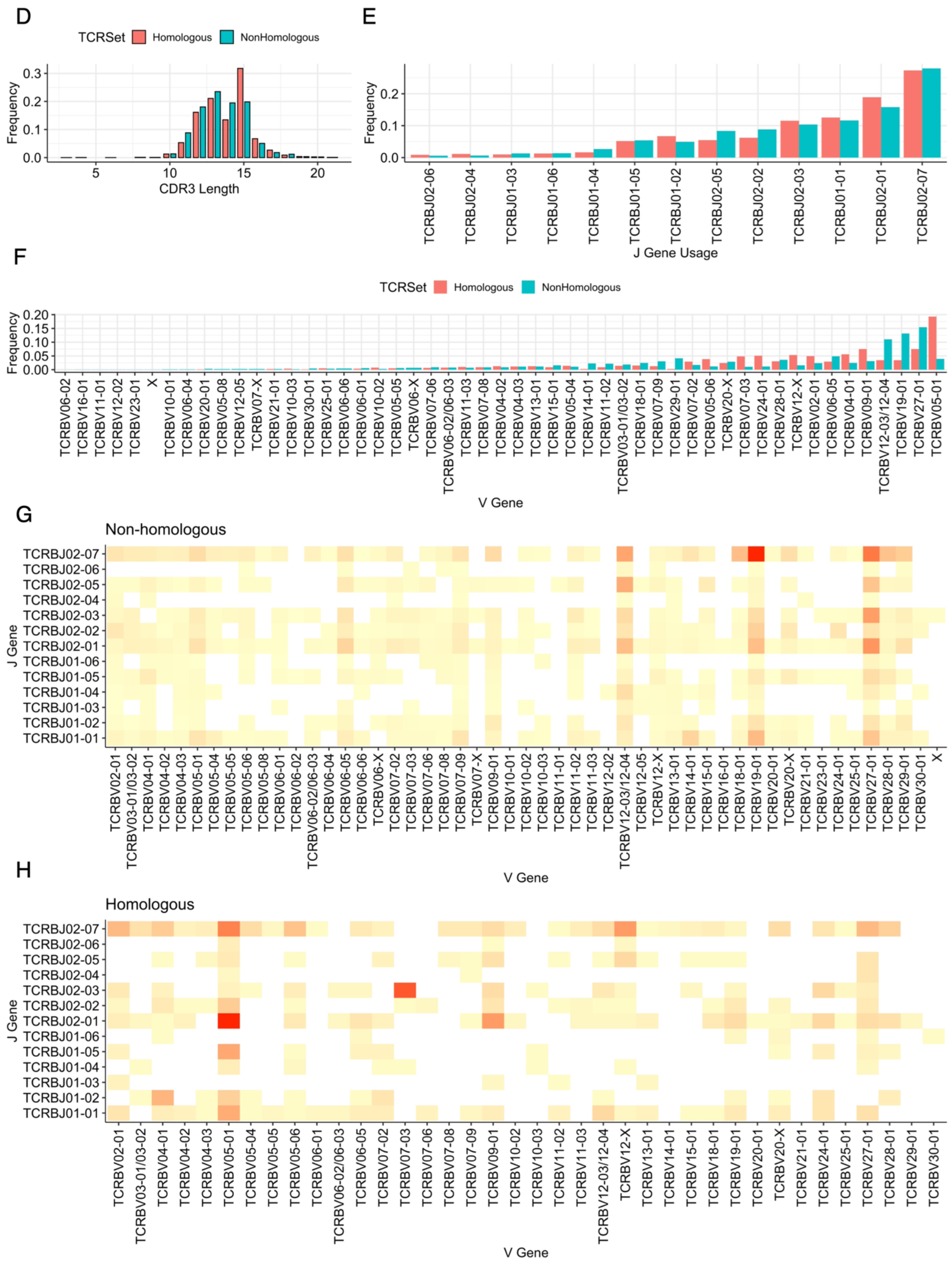

Supplementary Figure 5: A-B) Sequence logo plots visualizing the position-weight-matrix for (aligned) public CDR3b sequences which recognize only A) SARS-CoV-2-HCoV-homologous peptides, B) SARS-CoV-2-Non-homologous peptides. C) Line plot contrasting the Shannon entropy score for the aligned public CDR3b sequences for the TCRs which recognize only SARS-CoV-2-HCoV-homologous peptides or SARS-CoV-2-Non-homologous peptides. Shannon entropy score provides a measure of amino acid diversity at each position. D) Barplots showing the frequency of CDR3 lengths of public CDR3b sequences which recognize only SARS-CoV-2-Non-homologous peptides (labelled in red, 'NonHomologous'), or recognize only SARS-CoV-2-HCoV-homologous peptides (labelled in blue, 'Homologous'). E-F) Barplots showing the frequency of E) J gene usage, F) V gene usage, for public TCRs which recognize only SARS-CoV-2-Non-homologous peptides (labelled in red, 'NonHomologous'), or recognize only SARS-CoV-2-HCoV-homologous peptides (labelled in blue, 'Homologous'). G-H) Heatmaps showing the V and J gene usage of public TCRs which recognize G) only SARs-CoV-2-non-homologous peptides, H) only SARs-CoV-2-HCoV-homologous peptides.

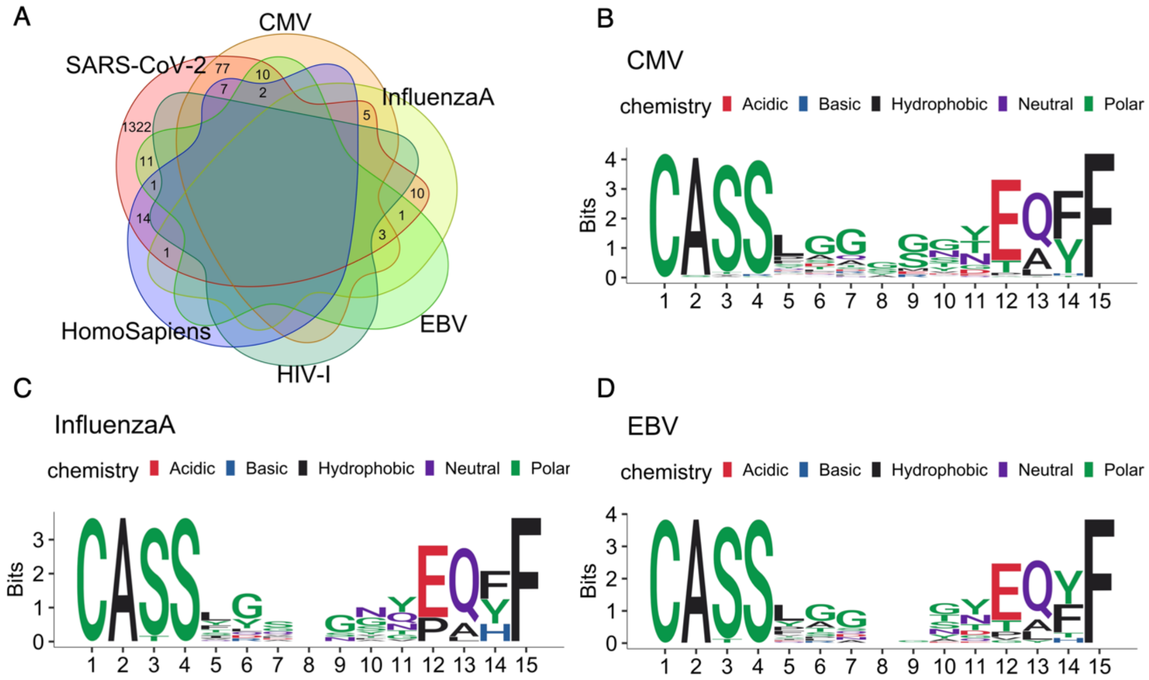

Supplementary Figure 6: A) Venn diagram showing the overlap between all unique SARS-CoV-2 CDR3b sequences from the MIRA dataset which recognize SARS-CoV-2-homologous peptides, and source antigens from VDJdb. B-D) Sequence logo plots showing the motifs of the SARS-CoV-2-specific CDR3b sequences which are also observed to recognize B) CMV, C) Influenza A, D) EBV.

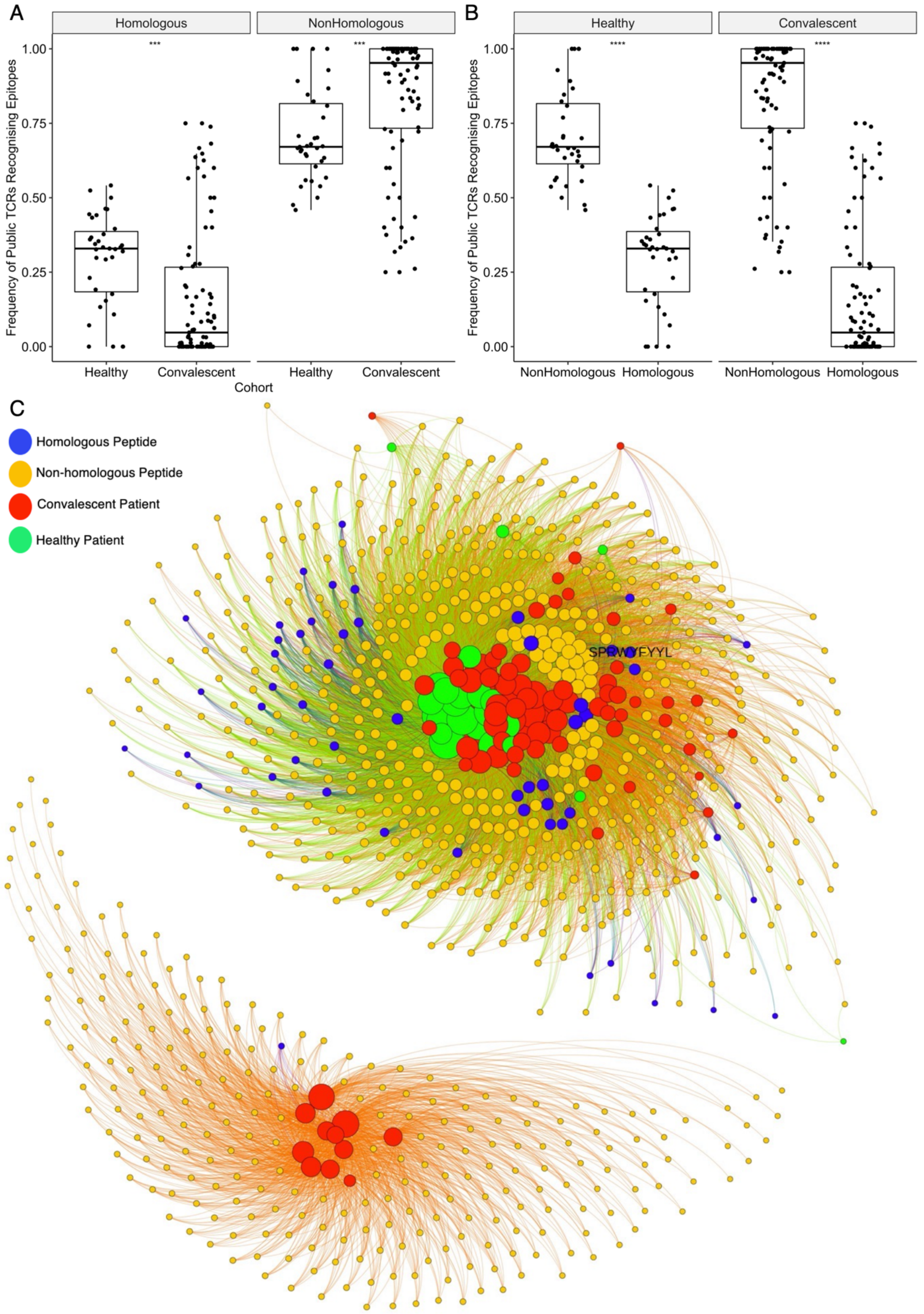

Supplementary Figure 7: A) Boxplot showing for each patient, the frequency of their public TCRs which recognise SARS-CoV-2 non-homologous or homologous SARS-CoV-2-HCoV peptides, grouped by non-homologous/homologous peptides. Comparing patient cohort. B) Boxplot showing for each patient, the frequency of public TCRs recognizing CoV-2 non-homologous or homologous HCoV-CoV-2 peptides, grouped by patient cohort. Comparing non-homologous/homologous epitopes. C) A bipartite graph showing the common-specificity of private TCRs recognizing SARS-CoV-2 homologous and non-homologous peptides. An edge between a patient and a peptide is observed if a patient possesses a private TCR recognizing that peptide.

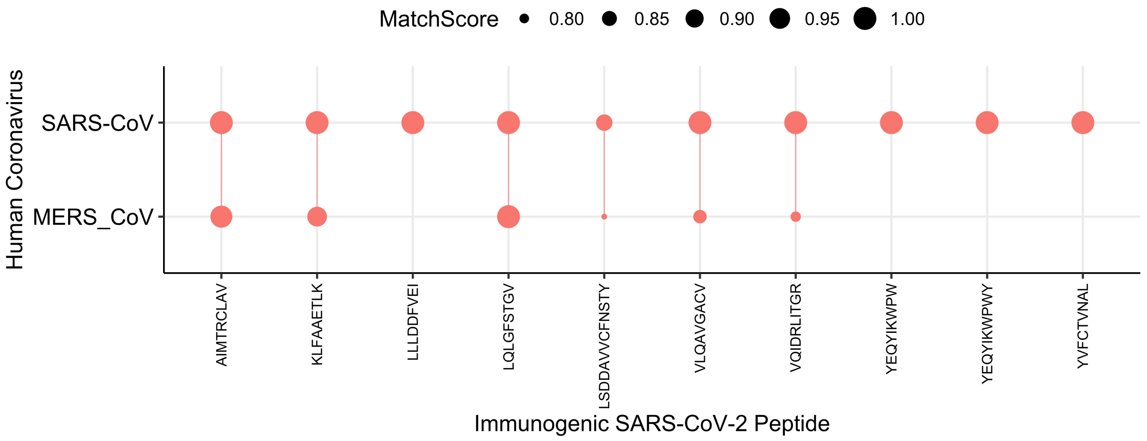

Supplementary Figure 8: A dot and line plot showing each SARS-CoV-2 peptide on the x-axis and to MERS or SARS-CoV if it exhibits a high similarity match. The size of each point reflects the MatchScore, i.e the similarity metric between the SARS-CoV-2 peptide visualisised and its counterpart match from SARS/MERS.

SARS-CoV-2 Peptide	Qualitative_Measure	HLA_Allele	HitSeq	MatchScore	HomologousEpitope	Length	SYMBOL	HitSeq_Binder	AASeq_Similarity
**RQLLFVVEV**	Positive	HLA class I	RQLLFVVDI	0.892	TRUE	9	GTPBP10	TRUE	0.778
**YIATNGPLK**	Positive	HLA-A*11:01	YIATQGPLK	0.884	TRUE	9	PTPRG	TRUE	0.889
**LLSAGIFGA**	Positive	HLA class I	LITAGIFGA	0.871	TRUE	9	SLC12A3	FALSE	0.778
**ALNTLVKQL**	Positive-Low	HLA-A*02:01	SLNTLLKQL	0.854	FALSE	9	CCDC169	TRUE	0.778
**GLTVLPPLL**	Positive,Positive	HLA-A*02:01,HLA-A*02:01	GLTVLPALL	0.851	FALSE	9	SLC2A4	TRUE	0.889
**SSRGTSPAR**	Positive	HLA class I	SSRDTSPAR	0.841	FALSE	9	CLASP1	TRUE	0.889
**LLFNKVTLA**	Positive,Positive	HLA class I,HLA-A*02:01	LLYNKMTLA	0.837	FALSE	9	MME	TRUE	0.778
**QEILGTVSW**	Positive,Positive	HLA-B*44:03,HLA-B*44:03	QEVLGSMSW	0.833	FALSE	9	NWD1	TRUE	0.667
**SPRRARSVA**	Positive	HLA-B*07:02	SPRRSRSIS	0.833	FALSE	9	SRSF7	TRUE	0.667
**AIMTRCLAV**	Positive	HLA class I	AVLTRCLVV	0.828	TRUE	9	LSM14B	TRUE	0.667
**LALLLLDRL**	Positive,Positive	HLA-A*02:01,HLA-A*02:01	LALALLDRI	0.821	FALSE	9	NUP205	TRUE	0.778
**FLLPSLATV**	Positive,Positive,Positive	HLA-A*02:01,HLA-A*02:01,HLA-A*02:01	FLIPSLAAI	0.819	FALSE	9	OR2AG1	TRUE	0.667
**RLFRKSNLK**	Positive,Positive,Positive	HLA class I,HLA-A*31:01,HLA-A*03:01	RLFKKSNIR	0.818	FALSE	9	GPR87	TRUE	0.667
**NLNESLIDL**	Positive,Positive-Low,Positive	HLA class I,HLA-A*02:01,HLA-A*02:01	NVNQSLLDL	0.814	FALSE	9	FTHL17	FALSE	0.667
**SELLTPLGI**	Positive	HLA-B*40:01	SELLKPLGL	0.814	FALSE	9	MBD4	TRUE	0.778
**EAFEKMVSL**	Positive-Low	HLA-B*08:01	EEFEKLVSL	0.81	FALSE	9	BHLHB9	TRUE	0.778
**RLDKVEAEV**	Positive	HLA class I	RLDSMEAEV	0.81	FALSE	9	CCDC151	TRUE	0.778
**STAALGVLM**	Positive	HLA-A*26:01	STAALAVLL	0.806	FALSE	9	CCL3	TRUE	0.778
**STAALGVLM**	Positive	HLA-A*26:01	STAALAVLL	0.806	FALSE	9	CCL3L1	TRUE	0.778
**VPHISRQRL**	Positive	HLA-B*07:02	VPHVSKERI	0.804	FALSE	9	KNL1	TRUE	0.556
**EYVSQPFLM**	Positive	HLA-A*24:02	EYIEKPFLM	0.8	FALSE	9	TTLL7	TRUE	0.667
**LLLDRLNQL**	Positive,Positive-Low,Positive-Low,Positive-Low,Positive,Positive	HLA-A*02:01,HLA-A*02:01,HLA-A*02:01,HLA-A*02:01,HLA-A*02:01,HLA-A*02:01	LLLDRVNDL	0.8	FALSE	9	DNAH5	TRUE	0.778
**STNVTIATY**	Positive,Positive	HLA-A*01:01,HLA-A*32:01	STHVTISTY	0.8	FALSE	9	RIOX2	TRUE	0.778
**VLKGVKLHY**	Positive	HLA-A*29:02	VLKGSKLHF	0.796	FALSE	9	KIF14	TRUE	0.778
**GMSRIGMEV**	Positive-Low,Positive,Positive,Positive	HLA-A*02:01,HLA-A*02:01,HLA-A*02:01,HLA-A*02:01	GMSRLGEEV	0.795	FALSE	9	EXD2	TRUE	0.778
**MASLVLARK**	Positive	HLA-A*68:01	MASLIVARQ	0.795	TRUE	9	SLC24A3	FALSE	0.667
**MASLVLARK**	Positive	HLA-A*68:01	MASLIVARQ	0.795	TRUE	9	SLC24A4	FALSE	0.667
**VLQAVGACV**	Positive	HLA class I	VLEAVGSCL	0.795	TRUE	9	ATM	TRUE	0.667
**KQEILGTVSW**	Positive,Positive	HLA-B*44:02,HLA-B*44:03	KQEVLGSMSW	0.849	FALSE	10	NWD1	TRUE	0.7
**SQASSRSSSR**	Positive	HLA class I	SRSSSRSSSR	0.837	TRUE	10	CLASRP	TRUE	0.8
**SQASSRSSSR**	Positive	HLA class I	SRASSRASSR	0.837	TRUE	10	GJA1	TRUE	0.8
**IVGVALLAVF**	Positive	HLA class I	ILGVVLLAIF	0.818	FALSE	10	CD163	FALSE	0.7
**TNVLEGSVAY**	Positive	HLA-B*35:01	TNLLEGAVAF	0.804	FALSE	10	AUP1	TRUE	0.7
**IEYPIIGDEL**	Positive	HLA-B*40:01	VEYPLIEDEL	0.796	TRUE	10	DNAH11	TRUE	0.7
**VENPDILRVY**	Positive	HLA-B*44:02	VESPKILRVY	0.792	TRUE	10	F11	TRUE	0.8
**ILPDPSKPSK**	Positive	HLA class I	ILPDPDDPSK	0.789	FALSE	10	ZNF592	TRUE	0.8
**KVAGFAKFLK**	Positive	HLA-A*11:01	SVAGFSRFLK	0.784	FALSE	10	FBXL4	TRUE	0.7
**FTISVTTEIL**	Positive	HLA class I	FTIRVTSEVL	0.783	FALSE	10	PNPT1	TRUE	0.7

Table S1: Peptides identified with high similarity to human proteome. HitSeq shows the match from the self proteome. HomologousEpitope shows whether the peptide is a SARS-CoV-2-HCoV homologous peptide or not. SYMBOL shows the gene from which the hit peptide is derived. HitSeq_Binder shows whether the hit peptide is predicted to bind an HLA allele. AASeq_Similarity shows the proportion of amino acids conserved between the SARS-CoV-2 peptide and the human proteome match.

Experiment	Cohort	Age	Gender	HLA-B	TCR	Peptide
**eAV91**	Healthy (No known exposure)	31	M	B*07:02	CASSELPGPPGEQYF+TCRBV0201+TCRBJ0207	LSPRWYFYY
**eAV91**	Healthy (No known exposure)	31	M	B*07:02	CASSELPGPPGEQYF+TCRBV0201+TCRBJ0207	SPRWYFYYL
**eXL31**	Healthy (No known exposure)	28	M	B*07:02	CASTLAGGPYNEQFF+TCRBV0501+TCRBJ0201	LSPRWYFYY
**eXL31**	Healthy (No known exposure)	28	M	B*07:02	CASTLAGGPYNEQFF+TCRBV0501+TCRBJ0201	SPRWYFYYL

Table S2: Two previously reported private TCRs are identified in additional HLA-B*07:02+ individuals at beta chain resolution, indicating these are cross-reactive public TCRs.

Supplementary Data File description

1: Data file containing each of the 126 SARS-CoV-2 peptides which map to 285 targets from HCoV. ‘Peptide’ shows the SARS-CoV-2 peptide, ‘HitSeq’ is the match from HCoV.

2: Data file containing the full set of SARS-CoV-2 peptides which have high similarity to the human proteome. ‘Peptide’ shows the SARS-CoV-2 peptide, ‘HitSeq’ is the match from the human proteome.

3: Data file containing the SARS-CoV-2-specific public TCRs which recognise SARS-CoV-2-HCoV-homologous peptides, which are also observed to recognise epitopes from other pathogens.

4: High resolution data file of Fig 5A.

5: High resolution data file of Fig 6B.

6: Data file containing a summary of the peptides recognized by public TCRs in the PubTCR-Homologous and PubTCR-Non-homologous groups, supplemented with those recognized in a sampled set of healthy patients. File contains a 1 or a 0 demonstrating whether a public TCR in each group of patients recognizes the peptide.

7: Data file containing the information from data file 3, but also includes information pertaining to whether key class I HLA alleles are observed in a patient with a public TCR recognizing each peptide.

8: Data file containing cohort information regarding the peptides most commonly recognized by private TCRs in the MIRA dataset.

9: Data file reporting the private TCRs which recognize the SARS-CoV-2-HCoV peptides shown in data file 5.

10: Data file containing information from Figure 6, exhibiting the peptides with hits to >=3 HCoV strains. File provides detailed information regarding SARS-CoV-2 peptide and the corresponding hit to HCoV.

11: Data file contains every TCR from the IEDB and the MIRA datasets which we used in this analysis, mapped to the recognized SARS-CoV-2 peptide.
